# Ularcirc: visualization and enhanced analysis of circular RNAs via back and canonical forward splicing

**DOI:** 10.1093/nar/gkz718

**Published:** 2019-08-22

**Authors:** David T Humphreys, Nicolas Fossat, Madeleine Demuth, Patrick P L Tam, Joshua W K Ho

**Affiliations:** 1 Victor Chang Cardiac Research Institute; 2 University of New South Wales, Sydney, Australia; 3 Embryology Unit, Children's Medical Research Institute; 4 School of Medical Sciences, Faculty of Medicine and Health, University of Sydney, Sydney, Australia; 5 School of Biomedical Sciences, Li Ka Shing Faculty of Medicine, The University of Hong Kong, Hong Kong

## Abstract

Circular RNAs (circRNA) are a unique class of transcripts that can only be identified from sequence alignments spanning discordant junctions, commonly referred to as backsplice junctions (BSJ). Canonical splicing is also linked with circRNA biogenesis either from the parental transcript or internal to the circRNA, and is not fully utilized in circRNA software. Here we present Ularcirc, a software tool that integrates the visualization of both BSJ and forward splicing junctions and provides downstream analysis of selected circRNA candidates. Ularcirc utilizes the output of CIRI, circExplorer, or raw chimeric output of the STAR aligner and assembles BSJ count table to allow multi-sample analysis. We used Ularcirc to identify and characterize circRNA from public and in-house generated data sets and demonstrate how it can be used to (i) discover novel splicing patterns of parental transcripts, (ii) detect internal splicing patterns of circRNA, and (iii) reveal the complexity of BSJ formation. Furthermore, we identify circRNA that have potential open reading frames longer than their linear sequence. Finally, we detected and validated the presence of a novel class of circRNA generated from *ApoA4* transcripts whose BSJ derive from multiple non-canonical splicing sites within coding exons. Ularcirc is accessed via https://github.com/VCCRI/Ularcirc.

## INTRODUCTION

High throughput sequencing is a technology regularly used to identify and quantitate transcripts that are present in enriched (polyA) or depleted (ribominus) RNA purifications. Circular RNAs (circRNA) are a class of abundant transcripts that can be identified in most library preparations that are not geared towards poly(A) enrichment. The use of RNase R, which selectively degrades linear RNA (1), is currently the only method that is utilized to selectively enrich for intact circRNA transcripts (2). However, with sufficient sequencing depth, circRNA can be detected in standard ribosome depleted libraries (3–5). Additionally, circRNA can also be detected in specialized library preparations designed to capture specific RNA fractions (e.g. polysome, RNA immunoprecipitations) (6–8).

CircRNA are identified from sequence data that capture the junction of discordant exons, commonly referred to as a backsplice junction (BSJ). The BSJ comprises a canonical splice donor and an upstream splice acceptor which may be derived from the same exon (single exonic circRNA) or any upstream exon of a linear transcript (multi-exonic circRNA). Tens of thousands of circRNA have been identified from a wide range of animals and plants and catalogued in online databases (9–12). Many have been identified to be tissue specific and/or developmentally expressed. The lack of a 5′ or 3′ RNA termini provides significant stability to the circRNA and their expression in diseases has found use as biomarkers (13,14).

Proposed mechanisms of circRNA biogenesis involve proximal localization of backsplice junctions via lariat formation or through base pairing of inverted complementary repeat sequences (2,4,15). The later mechanism has been demonstrated through the use of mini transgenes (16) and is regulated post transcriptionally by ADAR enzymes which alter the nucleotide composition and thereby influence the stability of hybridization (17). A number of splicing factors are also capable of influencing circRNA formation, which include Quaking (18), Rbm20 (19) and Mbln (20,21). Quaking, an important factor in EMT, binds to motifs within intronic sequences and dimerises bringing backsplice junctions into close proximity (18). The exact mechanism of how Mbln and Rbm20 regulate circRNA formation is yet to be elucidated but the fact that they influence promiscuous splicing patterns suggests that they have the capability to influence intronic interactions either through lariat formation or possibly a motif-based interaction.

The regulation of circRNA biogenesis and tissue specific expression patterns suggests that circRNA are not by products of splicing events. Functional roles for circRNA are largely unknown but there is increasing evidence that some circRNA can act as regulatory elements by decoying RNA or protein molecules. Specifically, circRNA can bind miRNA through multiple complementary binding sites to one or more miRNAs (3,22). Similarly, sequencing motifs within a circRNA can also interact with RNA binding proteins sequestering them from other activity (20,23).

CircRNA can also be translated. *In vitro* models have demonstrated that circRNA with internal IRES elements are capable of translating reporter genes (18). More recently ribosomal footprinting and mass spectrometry studies also support translation of circRNA (24). The absence of a cap and a poly(A) tail means that circRNA translation must be non-canonical. N6-methyladenosine (m6A) residues are enriched in circular RNAs and m6A modifications has been shown to be a possible driver of cap-independent translation (25).

The growing interest in circRNA coincides with the development of specific bioinformatic algorithms that focus on detecting, quantitating and annotating reads that capture backsplice junctions. Recent reviews of circRNA software reveal that all algorithms are effectively capable of detecting BSJ but vary in their ability to filter out false positives (26–28). However, to confidently identify and analyse circRNA, further downstream analyses such as internal exon usage, comparison of BSJ to parental FSJ expression, ORF and binding motifs identification, as well as tools for splice junction visualization are necessary. At this juncture, these complementary analyses were commonly performed in-house using custom bioinformatic scripts. To our knowledge, there is only two publicly available software tools that go beyond the detection of BSJ to characterize the internal exon use of circRNA, CIRI-AS/FULL (29,30) and FUCHS/circtools (31,32). There is therefore a void of software tools available to the community for comprehensive identification, analysis and visualization of circRNA. With this in mind, we developed the software package: Ularcirc.

Ularcirc combines BSJ detection with other analytical tools: It quantitates BSJ in relation with forward splice junction (FSJ) and the usage of internal exons. Ularcirc has an intuitive graphical interface menu system allowing the user to navigate to genes/junctions of interest and ultimately generate dynamic integrated genomic visualizations of both BSJ and FSJ. Furthermore, Ularcirc provides preliminary functional analysis by identifying open reading frames and miRNA binding motifs. Ularcirc is an open source software package written in R language and built using a shiny framework (http://shiny.rstudio.com). Ularcirc is dependent on Bioconductor databases and packages enabling it to be easily setup for a range of conventional and emerging model organisms. Using Ularcirc, we analysed our own data and a number of publicly available data sets to demonstrate its ability to discover and display novel processing and functional features of circRNA.

## MATERIALS AND METHODS

### Public datasets and alignment

All publicly available datasets analysed in this study (listed in Supplementary Table S1) were downloaded from NCBI sequence read archives using sratoolkit.2.8.2 fastq-dump. Datasets were trimmed for adaptor sequence and poor quality sequence using trimmomatic (33) using the following options LEADING:3 TRAILING:3 SLIDINGWINDOW:4:15 MINLEN:33. All data sets were aligned with the STAR aligner against either hg38 or mm10 genome using the two Pass star alignment protocol (34). We compared STAR aligner version 2.5 against 2.6 and confirmed that the newer release is far superior (Supplementary File 1). The second pass STAR 2.6 options were set to capture chimeric reads.

–chimSegmentMin 15 –chimJunctionOverhangMin 15 –outSAMtype BAM SortedByCoordinate –outSAMattributes All –chimScoreDropMax 37 –peOverlapNbasesMin 5 –chimMultimapNmax 1 –quantMode GeneCounts.

We note that the –chimSegmentMin is within the range of what other circRNA detection software recommends. For example, at this point in time, the websites for circExplorer (16) and DCC (35) recommends the –chimSegmentMin to be 10 and 20 respectively.

Some data sets were also aligned with BWA aligner (VN:0.7.17-r1188) using bwa-mem using 8 threads and setting minimum output scores to 19 (-T 19).

### Implementation of Ularcirc

Ularcirc is written in R using the shiny framework and is designed to work on any standard personal computer that can run R. Ularcirc software should be installed as described on the Ularcirc github page (https://github.com/VCCRI/Ularcirc). For full functionality Bioconductor genome and transcriptome databases relevant to a project should be downloaded prior to starting Ularcirc. There are numerous functional aspects built into Ularcirc, a detailed manual and various screen casts (available on github website) have been prepared to demonstrate their use. Below are descriptions of the implementation of key Ularcirc functions.

#### Data import

Ularcirc can import files generated from STAR, CIRI and circExplorer. Aligned data can be stranded or unstranded. The STAR aligner generates three output file that can be imported into Ularcirc, these include: (i) chimeric output file (file extension ‘Chimeric.out.junction’), (ii) canonical splice junction output file (file extension ‘SJ.out.tab’) and (iii) the gene count files (file extension ‘ReadsPergene.out.tab’). Output from CIRI2 (file extenson ‘.ciri’) and circExplorer2 (file extenson ‘.ce’) can also be uploaded. Ularcirc identifies and groups sample output files by the filename prefix. Consider the following hypothetical example that contains two samples each with five input data files. Ularcirc extracts sample ID from the file prefix (i.e. ‘Hela_untreated’ and HeLa_condA’).

**Table:** 

Sample	Output file naming
HeLa untreated	HeLa_untreated.Chimeric.out.junction
	HeLa_untreated.SJ.out.tab
	HeLa_untreated.ReadsPerGene.out.tab
	HeLa_untreated.ciri
	HeLa_untreated.ce
HeLa conditionA	HeLa_condA.Chimeric.out.junction
	HeLa_condA.SJ.out.tab
	HeLa_condA.ReadsPerGene.out.tab
	HeLa_condA.ciri
	HeLa_condA.ce

#### CircRNA sequence assembly

Predicted circRNA sequences are assembled from intersecting exons of the selected gene model. Intersecting exons are defined as the longest combination of one or multiple exons from a single transcript that match within coordinates of the backsplice junction. Additionally, the splice donor and acceptor junctions that define a BSJ must align to a splice donor and splice acceptor of the concatenated transcript exons. The identified exonic coordinates are then used to extract genomic sequence from the matched genome reference.

#### Backsplice sequence assembly

The same process as for total circRNA sequence assembly with 50nt of sequence each side of the backsplice junction displayed. The backsplice junction is therefore positioned in the middle of the displayed sequence and is highlighted by the inserted ‘.’ character.

#### ORF detection

Open reading frames were calculated from circRNA sequence (as defined under subheading ‘circRNA sequence assembly’) by concatenating the sequence for a total of three times. Amino acids from the three possible open reading frames were calculated using the ‘translate’ function from the Bioconductor biostrings function (36). The longest open reading frame extracted is reported.

#### miRNA binding predictions

Ularcirc queries the R Bioconductor database miRbase.db (37) which is assembled from miRBase (38) and extracts the reverse complement of miRNA seed sequences. Seed sequences of a user defined length (6–8 nt) are matched against the selected circRNA candidate and are displayed in a circus like plot and tabulated.

#### RNA alignment distribution (RAD) score

**Figure 1.**
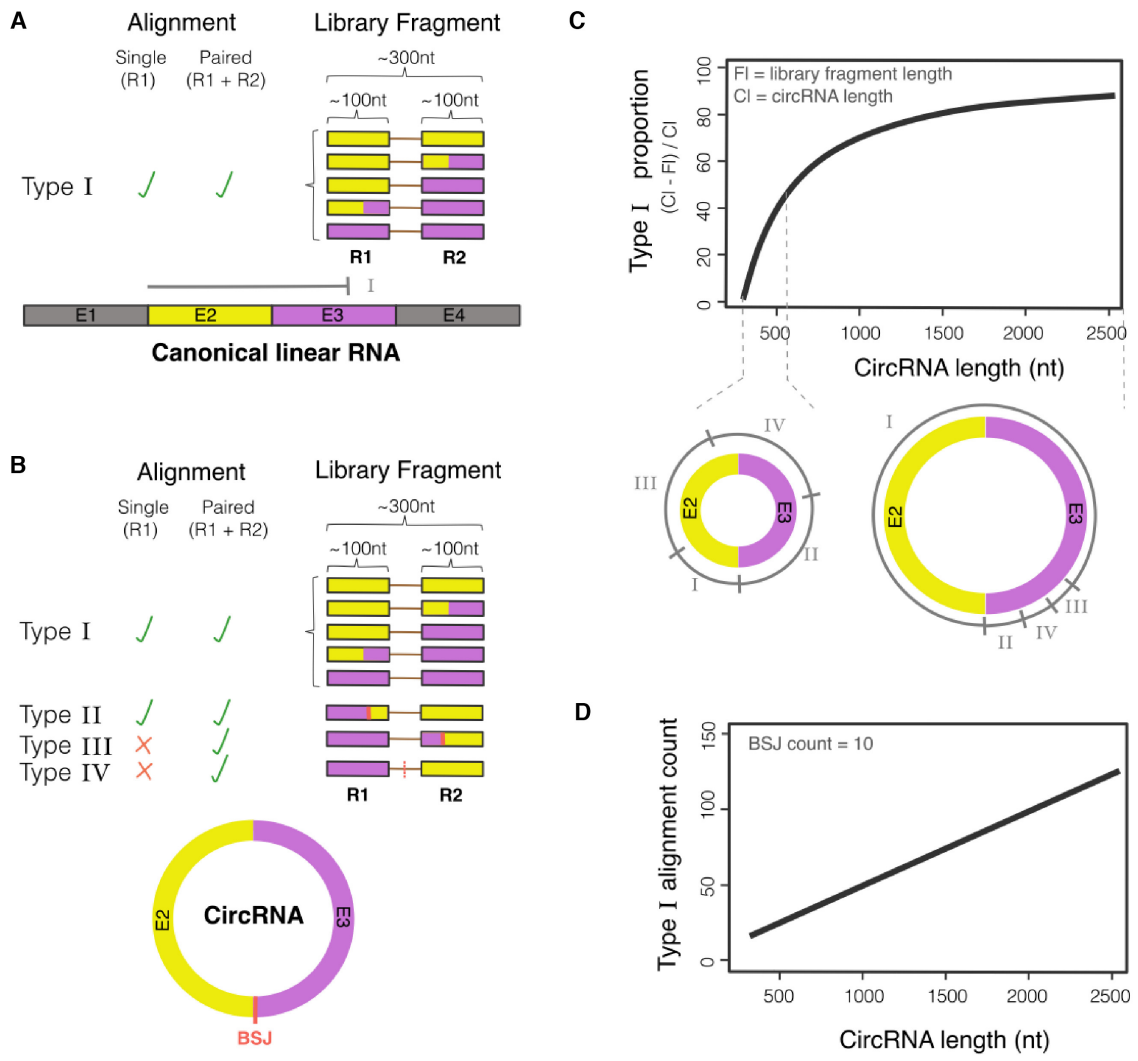
Theoretical modelling of sequence alignments from circRNA and linear RNA. (**A**) Type I alignments are those that are indistinguishable between linear and circRNA. (**B**) BSJ of circRNA are detected from type II and III alignments and can be inferred from type IV alignments. (**C**) The proportion of type I alignments increase with circRNA size. The small and large circRNA schematics below the graph highlight the different proportion of alignment types across a small and large circRNA. The alignment ranges labelled I through to IV defines the 5′ start position of the aligned read pair. (**D**) Inferred number of type I alignments from a different size range of circRNA that have a theoretical BSJ count of 10.

We define type II and type III alignments as paired end sequence reads that capture BSJ in either the forward or reverse read pair (Figure 1B). The RAD score is calculated from chimeric.out.junction files generated from STAR aligned data. The proportion of type II and type III alignments are calculated from match lengths annotated in CIGAR strings provided for each segment of detected chimeric junctions. Note that the longest match effectively identifies which read does NOT capture the BSJ. We tested this algorithm on synthetically type II, type III and type IV alignments (refer to Supplementary File 1) generated from 1834 true positive backsplice junctions as defined in circRNA software reviews (27,28). These synthetic data sets enable us to define the ground truth and track how reads were categorized. The algorithm defined above successfully identified >99% of type II and type III alignments.

The RAD score does not effectively work on BSJ that have a low count as small changes in read assignments will cause significant skewing. Therefore, in Ularcirc, we have set the default threshold to provide a RAD score at 10 reads (only applies for STAR aligned data). To avoid confusion any BSJ that has a count <10 will not be assigned a RAD score value. When assessing the FSJ support score for the 1834 circRNA, an average RAD score from both ribominus data sets was calculated (Figure 3).

#### Forward splice junction (FSJ) support score

This identifies donor and acceptor splice junctions that are common to both FSJ and BSJ. If the donor splice junction of a BSJ is detected to be used in a FSJ, a value of 1 is added to the FSJ score. Similarly, if the acceptor splice junction of a BSJ is detected to be used in a FSJ, a value of 1 is added to the FSJ score. Therefore, each BSJ can have a FSJ score of either 0, 1 or 2. When assessing the FSJ support score for the 1834 circRNA, an average FSJ support score from both ribominus data sets was calculated (Figure 3). The FSJ support score is not suitable for RNaseR data sets as linear isoforms are degraded.

#### CircRNA annotation

This is an optional feature for STAR aligned data. When selected, all BSJ are annotated with overlapping potential parental genes names that are identified from the selected transcript data base. Importantly, there is no requirement for BSJ to overlap with exon boundaries.

### CircRNA biogenesis analysis (transcript derived vs transcript by-product)

This analysis assesses if circRNA are by-products or directly derived from linear parental transcripts. Parental transcripts of circRNA are identified as those having identical exon boundaries to that of the circRNA. CircRNA derived from spliced out exons of parental transcript are referred to as transcript by-products, whereas circRNA derived from exons common to linear transcript (i.e. not spliced-out exons) are defined as transcript derived.

For each parental transcript, splice junctions that span across exons of circRNA were quantitated in Ularcirc by annotating tables with the ‘Annotate with FSJ coverage’. This feature returns tables with raw counts of BSJ and FSJ broken down to three categories: ‘internal’, (average FSJ counts that occur within boundary of circRNA), ‘canonical’ (average FSJ count outside boundary of circRNA), ‘spanning’ (FSJ that span the BSJ of circRNA). The spanning junctions were normalized against the corresponding BSJ counts.

### Sensitivity and specificity of circRNA software

Sequencing reads from two ribominus and two ribominus + RNaseR treated data sets were aligned with either STAR (2.6) or BWA (39). For BWA aligned data BSJ count tables were generated from BAM files using CIRI2 (v 2.0.6) and circExplorer2 (v2.3.3) software. For STAR aligned data BSJ count tables were generated from Chimeric junction output files using Ularcirc and circExplorer2 software. We performed sensitivity and specificity analysis at two different BSJ minimum count thresholds: (i) only considering BSJ that were present in at least one ribominus data set having at least 3 counts, (ii) only considering BSJ that were present in both ribominus data sets having at least nine counts.

### Reconstruction of true positive BSJ/circRNA sequences

A previous study identified true positive candidates from matched ribominus and RNase R treated data sets and provided access via R code and data sets (4,28). We retrieved BSJ coordinates for these 1532 true positive circRNA and reconstructed BSJ sequences or complete predicted circRNA sequences using the Ularcirc stand-alone function bsj_fastq_generate() or bsj_to_circRNA_sequence() respectively. The bsj_fastq_generate() function allows specific positioning of the BSJ within paired end reads that are created with unique identifiers that allow easy assessment/recovery post alignment.

### Generation of murine small intestine epithelial cell RNA-Seq datasets

For the dataset of murine epithelial cells of the small intestine, we used 1 μg of total RNA extracted from wild type adult mouse as in (40) and generated the NGS library prep using the ‘TruSeq stranded total RNA sample preparation’ kit from Illumina strictly following manufacturer instructions (with depletion of ribosomal RNA using the ‘RiboZero rRNA removal mix’). Library was sequenced using an Illumina 2500 and fastq files were provided by the precinct sequencing provider.

### RNA RT-PCR

Total RNA was obtained from epithelial cells of the small intestine isolated as described in (40) or from small intestine of human biopsies (Clontech). For Figure 6B, C, D, E, H and I, 1 ug of total RNA was reverse transcribed. For Figure 6G, 50 ug of total RNA was treated with RNAseR (Epicentre) according to manufacturer instruction, then purified using RNeasy Mini kit (Qiagen) following manufacturer instruction. 100 ng of RNAseR-treated RNA was then reverse transcribed in parallel of 100 ng non-treated RNA for comparison. Reverse transcription was performed using the SuperScript III First-Strand Kit (Invitrogen) and following manufacturer instructions with random hexamers (Figure 6B, C, D, E, G and H) or oligo(dT)_20_ (Figure 6G) provided with the kit. 1–10 ul of cDNA (undiluted or diluted 1:10) was used as input for PCR amplification with Biomix Red (Bioline). Conditions of PCR are 95°C for 30 s, 60°C for 30 s and 72°C for 30 s for 30 or 35 cycles. For Figure 6G, same inputs and PCR conditions were used for all the samples run on the same gel. The PCR products were visualized on TAE 2% agarose gel.

Sequence of *ApoA4* primers used in Figure 6 are listed below:

- a mouse: 5′-CCTGCAGTCAATCTGCACA
- b mouse: 5′-AGTGACATCCGTCTTCTGAA
- c mouse: 5′-GATGCTAGTACGTATGCTGA
- b mouse for HTS: 5′-CCTCTCTATGGGCAGTCGGTGATAGTGACATCCGTCTTCTGAA
- c mouse for HTS: 5′-CCATCTCATCCCTGCGTGTCTCCGACTCAGGATGCTAGTACGTATGCTGA
- d mouse: 5′-GCTTAGCTGGGTAAACGTAG (15 first nt complementary to region in exon 2; last 5 nt complementary to region in exon 3)
- a human: 5′-TGTAGGGAGGATCCAGTGT
- b human (for PCR with a human): 5′-CCTGGTCAGCACTGACCT
- b human (for PCR with c human): 5′-AGTTGCTGGGTGAGTTCAGA
- c human: 5′-TCTTCCAGGACAAACTTGGA
- b human for HTS: 5′-CCTCTCTATGGGCAGTCGGTGATAGTTGCTGGGTGAGTTCAGA
- c human for HTS: 5′-CCATCTCATCCCTGCGTGTCTCCGACTCAGTCTTCCAGGACAAACTTGGA

### Sanger sequencing

Products i and ii amplified by PCR Figure 6E were isolated and purified from gel with Wizard kit (Promega) and send for Sanger sequencing with primer c mouse to the Australian Genome Research Facility (AGRF).

## RESULTS

### Classification and utility of reads aligned to circRNA

We classified four different sequence alignments for circRNA from data sets generated with current short read technologies and refer to these as Type I–IV (Figure 1A and B). Type I alignments are those that cover nucleotides that span regions within a single exon or across multiple concordant exons. These nucleotides while within a circRNA are indistinguishable to positions of a linear RNA and cannot be used for circRNA identification. Type II and III are alignments that capture a BSJ in either the primary or paired end read, respectively and can be used as direct evidence of a circRNA existence. Type IV are discordant alignments of paired end reads that flank but do not contain a BSJ. On their own, type IV alignments do not identify a BSJ and therefore should only be used as supporting evidence.

We calculated the theoretical distribution of Type I–IV alignments and find that there is an increasing proportion of Type I alignments with circRNA size (Figure 1C, materials and methods). These calculations have been constructed on typical features of short read RNA-Seq libraries, where the average library fragment size is ∼300 bp and paired end read length is 100nt (Figure 1B). Consequently, the proportion of type I alignments is quasi null for circRNA smaller than a library fragment length (i.e. 300nt) but increases steeply for circRNA having sizes up to twice that of a generated library fragment (i.e. 600nt where type I alignments ≤ 50%) because type I alignments are becoming feasible in this size range (Figure 1C). CircRNA that have a size greater than twice that of a library fragment (i.e. 600nt) result in a more gradual increase of type I-alignment proportion. Type I alignments from a circRNA are reported in the standard BAM alignment files and cannot be distinguished from fragments generated from linear RNA. Therefore, in situations where a circRNA is abundantly expressed relative to linear parental transcripts (>10%), it is very likely that type I alignment reads from the circRNA may contribute a significant proportion of aligned reads to the parental (pre-circularized/ native) transcript, which may impact on various downstream analysis. To demonstrate this, we calculated the theoretical number of type I alignments that are produced from different sized circRNA with an arbitrary fixed absolute BSJ read count of 10. We found that the number of type I alignments will be multiple of the BSJ count as the size of a circRNA increases (Figure 1D).

We reasoned that the relative abundance of type I alignments is likely to prove insightful, particularly when capturing a FSJ. Because of this, we incorporated into Ularcirc the ability to quantitate, filter and visualization FSJ from type I alignments. To our knowledge no other circRNA tools incorporates FSJ information from type I alignments. The closest is CIRI-AS which detects and quantitates FSJ detection from type II and type III alignments to resolve internal circRNA splicing (29). CIRI-AS approach limits detection of FSJ to be within the fragment length of the BSJ. Below, we describe insights we have gained from, in addition to BSJ detection from type II and III alignments, cross analysis of FSJ from type I alignments.

### Overview of the Ularcirc pipeline and interface

BSJ and FSJ can be captured in RNA-sequencing data through reads covering discordant and canonical junctions respectively. Several aligners are capable of correctly aligning reads that span FSJ, but very few implement the detection of BSJ. The STAR aligner has become one of the more popular RNA-Seq aligners due to its speed and accuracy (41,42). However, it is less well known that the STAR aligner can also provide output count tables for canonical and non-canonical ‘chimeric’ junction counts (independent of gene model). These count tables are simple tab delimited text files that are considerably smaller than traditional aligned BAM files and can be easily processed on desktop computers. We saw an opportunity to utilize this resource and designed Ularcirc to integrate FSJ and BSJ information by generating insightful visualizations, using the ‘Sushi’ genomic visualization library (43), and build detailed annotated count tables for in depth analysis.

**Figure 2.**
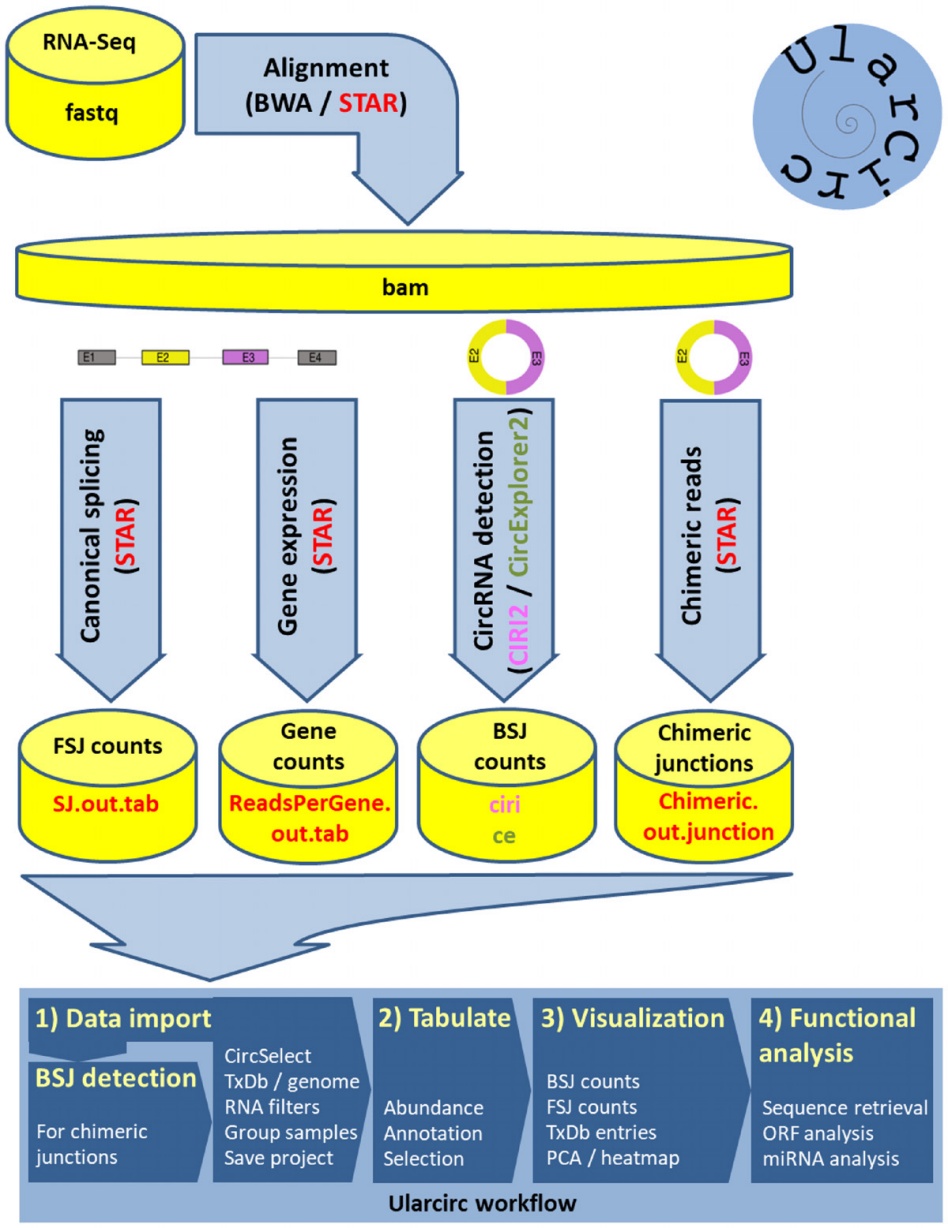
Schematic of Ularcirc file input dependencies and workflow. Sequence reads are aligned with either the STAR or BWA aligner. Users can utilize existing circRNA detection tools (CIRI2, circExplorer2) to generate BSJ count tables. Alternatively, chimeric junction tables generated by the STAR aligner can be directly uploaded into Ularcirc where inbuilt functions assemble BSJ count tables. Users can then interact with the assemble tables and build plots (e.g. heatmaps, PCA plots), visualize BSJ and FSJ and corresponding gene model, and retrieve sequence and analyse them for open reading frames and miRNA binding sites.

We also designed Ularcirc with the option to use the output provided by CIRI2 (44) and circExplorer2 (16), two reputable and heavily used circRNA software recognized for their performance in identifying circRNA (27,28). Both software pipelines produce one output file per sample that simply need to be renamed with the correct extension (see material and methods section) to be uploaded and analysed via Ularcirc (Figure 2). Nevertheless, Ularcirc should not be regarded as only a companion software for existing pipelines as it also provides a unique visualization interface and numerous original downstream analysis steps which, according to our knowledge, have not been implemented elsewhere at this point in time (Table 1). Furthermore, Ularcirc circRNA inbuilt detection pipeline works independently of gene models and therefore can reveal circRNA not identified by other software (see examples further below). For all these reasons, we believe that Ularcirc extends and improves the analysis of circRNA.

**Table 1.** Software features of Ularcirc, circExplorer and CIRI

	Ularcirc	circExplorer	CIRI
Interface	GUI	Command line	Command line
Gene model dependency	Y/N^a^	Y/N^b^	N
Multisample analysis	Y	N	N
Normalization	Y	N	N
Visualization	Y	N	N
PCA plot	Y	N	N
Heatmap	Y	N	N
circRNA sequence analysis	Y	N	Y

^a^Does not require gene model for assembling BSJ counts from STAR input. Is required for funtional sequence analysis.

^b^Not required for *de novo* discovery.

Ularcirc provides real time data analyses by dynamically generating an integrative genomic view (IGV) of BSJ and FSJ counts annotated with known transcript exon/intron boundaries. Junction count data is plotted as loops, where the loop height represents sequence counts and x-axis intersections represents the genomic coordinates of the splice junction. Junction data is overlaid onto a pre-existing gene model where transcript exons are graphed as rectangle blocks, similar to what is found in most genomic viewers (Supplementary Figure S1A). Ularcirc also provides a zoom functionality enabling resolution for dense and complex gene structures (Supplementary Figure S1B). Furthermore, any FSJ or BSJ can be selected from the raw data tables resulting in the corresponding splice junction in the IGV to be highlighted.

Ularcirc is written in R using the shiny framework and the application can be launched as a standalone program on individual machines or as a multi-user setup through a server or cloud installation. Ularcirc has an intuitive graphical user interface while the backend automatically detects installed Bioconductor resource databases and other libraries (Figure 2). As of Bioconductor release 3.5 there are 13 organisms with complete genome, annotation and transcript databases (Supplementary Table S2). Ularcirc is immediately applicable for all these organisms once the appropriate libraries are installed. Furthermore, Bioconductor has workflows for the incorporation of other genomes and annotation files, thereby making Ularcirc compatible with any organism.

### Quantitating, annotating, and quality filtering of circRNA

We designed Ularcirc to follow a simple four-step workflow that enables quick identification of putative circRNA (Figure 2). After data import, users can assemble a table of annotated circRNA from an individually selected (i.e. single count column) or a group defined (i.e. multi count column) data sets in a short timeframe using a standard desktop computer (Supplementary File 1). If output files from different external programs have been uploaded (e.g. CIRI2 or circExplorer2), users can select and switch between data sources for comparison.

**Figure 3.**
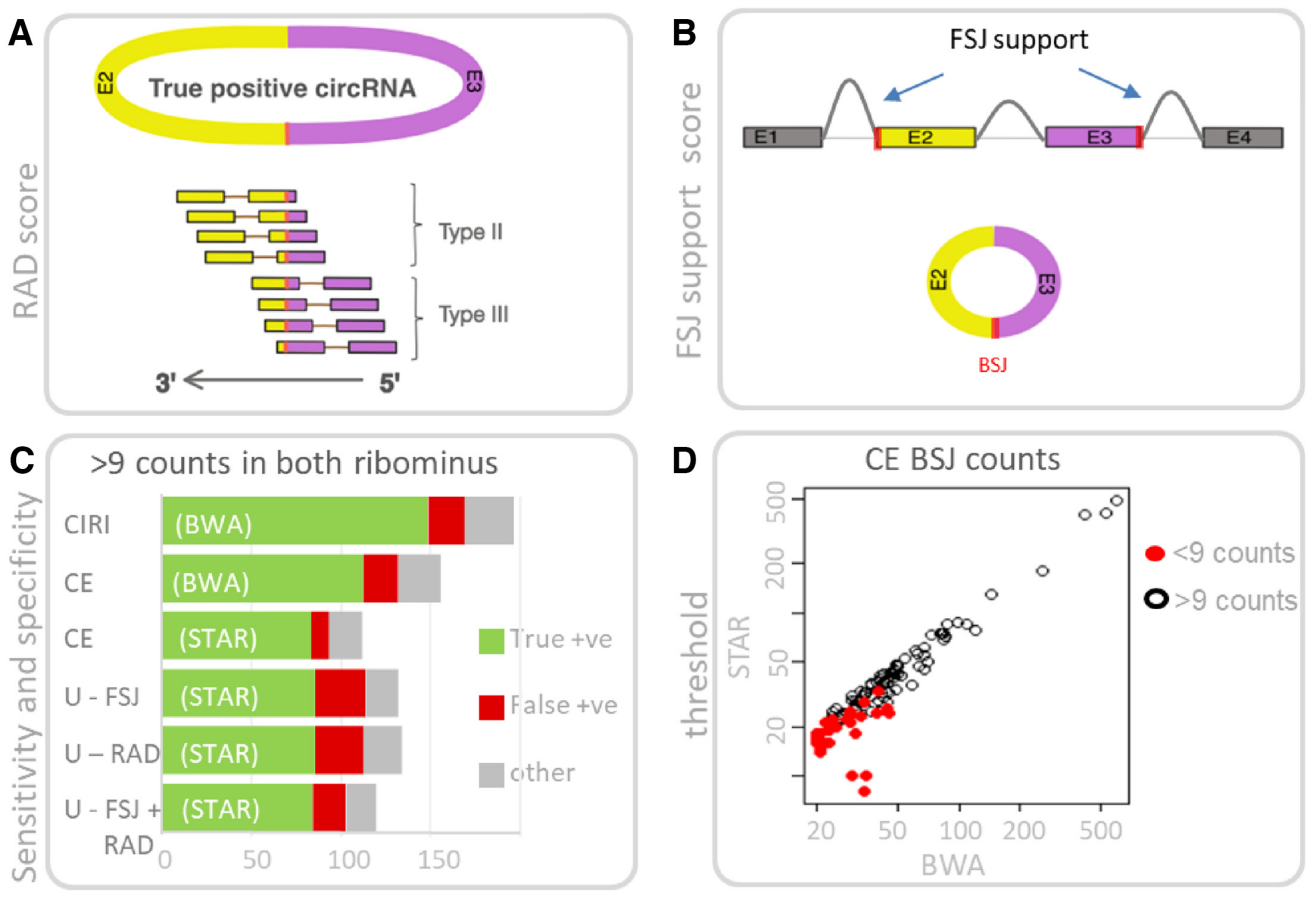
Alignment features that distinguish true and false positive circRNA. (**A**) True positive circRNA should display a spectrum of type II or type III alignments (refer Figure 1B). The RAD score is defined as (Type II)/(TypeII + Type III) and for true positive circRNA the RAD score should theoretically be close to 0.5. (**B**) The splice junctions that define a BSJ (red bars) are also utilized in canonical splicing. The FSJ support score calculates if the BSJ coordinates are also utilized in FSJ. For true positive circRNA the FSJ support score should theoretically be 2. (**C**) Performance of Ularcirc, CIRI2 and circExplorer2 software in recovering abundant BSJ from matched ribominus and RNaseR data sets. Abundant BSJ are defined as having minimum count of 9 in both ribominus data sets. (**D**) True positive circRNA detected by circExplorer2 (CE) from BWA aligned data are also detected in STAR aligned data. A proportion of candidates were not reported from STAR aligned data (red dots) because the minimum count threshold (>9 counts per ribominus data set) was not met.

Ularcirc has inbuilt algorithms that detect abundant BSJ directly from chimeric junction files of the STAR aligner without the need of a gene model. BSJ are quantitated from type II and type III alignments and can be validated against two novel metrics termed the read alignment distribution (RAD) and FSJ support scores. The RAD score is defined as the ratio of type II vs type III alignments (RAD = type II/(type II + type III)), and can only be applied to BSJ having a read count of at least 10 as significant skewing would occur at lower counts (Figure 3A). In theory, genuine circRNA should generate equal proportions of type II and III alignments and therefore have a RAD ratio approaching 0.5 (Figure 3A), but this may vary between circRNA depending on sequencing coverage biases. False positive circRNA are derived from homologous linear sequences of neighbouring exons (as identified in (26) Supplementary Figure S2A, B) and are not influenced from sequence coverage bias and therefore produce RAD scores close to either 0 or 1 (Supplementary Figure S2C).We therefore recommend filtering using a broad RAD score range that will keep many true positives and exclude false positives (e.g. include circRNA with RAD score >0.1 and <0.9).

The FSJ support score is an integer value between 0 and 2 which is calculated by adding a value of one to both the BSJ splice donor and acceptor if they are also identified to be utilized in canonical splicing (Figure 3B). We find that many RNase-R sensitive (i.e. false positive) circRNA produce a FSJ score of 0, which suggests that this is a good threshold to apply as a filter (Supplementary Figure S2D). The FSJ score is based on the knowledge that BSJ of a circRNA typically utilizes exon junctions of the parental transcript (4). Most circRNA pipelines benefit from this knowledge by filtering BSJ against existing gene models, and this approach has been proven effective in removing false positive candidates. However, this implementation limits their usefulness to organisms that have mature gene model assemblies. The ‘FSJ support’ metric implemented in Ularcirc is similar but the execution is different as it identifies exon boundaries via FSJ directly from the sequencing data, and not from a pre-determined gene model (Figure 3B). This negates the requirement of existing gene models and allows de novo discovery of circRNA.

Both RAD and FSJ support scores can be used independently or in combination within Ularcirc to filter out false positive candidates. We compared the Ularcirc inbuilt algorithm in detecting circRNA from matched RNaseR and ribominus datasets (4). These data sets have previously been used to assess circRNA discovery tools and only considered BSJ that were present in ribominus data with a minimum BSJ count of 3 (28). We repeated this analysis but limited analysis to BSJ that had a minimum count of 10 as this would allow us to assess the RAD filtered data which was only applicable to BSJ counts in this range. As a reference, we also analysed the same datasets with circExplorer2 (using either BWA or STAR aligner inputs) and CIRI2 (BWA aligner inputs) using the same count thresholds as these programs were highly rated in their performance (28). A greater number of true positives were identified from BWA aligned data (via CIRI and circExplorer pipelines) compared to STAR aligned data (circExplorer pipeline) (Figure 3C). Given that the same circExplorer pipeline was applied to both STAR and BWA aligned data, we suspected that this difference was due to the aligner. To test that, we generated 1532 synthetic type II and III BSJ containing 100nt paired end reads that were either overlapping or non-overlapping (see material and methods section). Data sets were aligned with recent versions of the STAR aligner. We identified that both versions of STAR did not detect BSJ in the first 20nt of the 5′ end of an aligned sequence (Supplementary File 1), which can be explained by the defined minimum chimeric length parameter (refer methods). We also confirmed that STAR version 2.6 resolved a previous issue where BSJ in overlapping reads were not detected (Supplementary File 1). It is very likely that the STAR aligner can be further optimized for BSJ detection as all true positive candidates that met the minimum count filter in BWA alignments were present in STAR aligned data but having lower read counts (Figure 3D). Furthermore, an essential benefit of using the STAR aligner over the BWA aligner is its ability to extract FSJ. Therefore, the STAR aligner is robust and more versatile than the BWA aligner in detecting circRNA.

We then compared Ularcirc RAD and FSJ support scores filtered outputs to circExplorer2 outputs, as the files generated by both software can be obtained with the same aligner, i.e. STAR. We defined true positive BSJ candidates as those that have a RAD score between 0.1 and 0.9. For abundant BSJ (i.e. >9 counts) applying the RAD score filter produced a list of BSJ of which 86 (64%) were classified as true positives, compared to circExplorer which identified 84 (Figure 3C). The RAD score was not as efficient as circExplorer in filtering out false positives (20.1% versus 8.9%). Interestingly many of the false positives appeared to be lariat RNAs as well as BSJ of circRNA validated in other studies—including the RNaseR sensitive circRNA CiRS-7 (data not shown). It is possible that many of these candidates are therefore indeed true positives and are perhaps linearized versions of bona fide circRNA transcripts. The FSJ support score performed similarly to the RAD score in identifying bona fide abundant BSJ (Figure 3C). Using both the RAD score and the FSJ support score improved the detection of false positives without impacting true positive discovery (Figure 3C). We conclude that the RAD and FSJ support scores are suitable for discovery of abundant circRNA (having BSJ count > 9) and that they should be used in this case or when no gene model are available and for *de novo* discovery. However, for low abundant circRNA analysis and when a comprehensive gene model is available, users should use other validated pipelines such as CIRI2 and circExplorer2.

### Visualization and analysis of circular and linear transcripts

Ularcirc has functionality to display and analyse transcript sequences from circRNA and linear transcripts. CircRNA sequences are generated by concatenating parental transcript exons sequences within the boundary of a selected BSJ. Users can select to display junction sequences from either a BSJ or FSJ, which enables easy primer design or downstream analysis. Additionally, Ularcirc has two functional analysis tools that can be used on a selected circRNA, which are (1) identification of the longest open reading frame (ORF) and (2) miRNA binding motif analysis.

The open reading frame analysis tool provides a circos-like plot representation of the longest open reading frame detected within a selected circRNA. The amino acid sequence derived from the ORF is also displayed enabling downstream analysis. It is known that some circRNA contain long ORF, for example the circRNA from Slc8a1 contains a long ORF that has been tentatively linked to a truncated translated product (45). Using Ularcirc, we identified a number of other abundant cardiac circRNA encoding significantly long ORF. Interestingly we identified circRNA where the ORF extends across the full circle past the initial start codon (Supplementary Figure S3). For example, the ORF identified within a circRNA derived from HipK3, a single circularized exon (1099 nt in size), can in theory produce a 388 amino acids product which is 22 amino acids longer than what is possible on an equivalent length linear transcript. Interestingly the BSJ from Hipk3 circRNA is one of the most abundant in the developing heart and has a similar count to canonical FSJ of the linear parental construct.

The other function analysis tool within Ularcirc identifies miRNA binding sites. Ularcirc provides functionality to visualize the locations of miRNA binding sites of a selected circRNA (refer methods). Target sites are identified from miRNA seed sequences whose start position and size can be defined by user. The limited complexity in a short miRNA seed sequence can result in many single hits. Ularcirc provides the capability of filtering miRNA binding sites that occurs at a minimum frequency. After filtering, the short-listed binding sites are presented as a circos-like plot and the specific miRNA and the number of binding sites tabulated (Supplementary Figure S4).

### Splicing analysis of linear transcript and circRNA biogenesis

We wanted to assess if the FSJ output generated by the STAR aligner was capable of reproducing known alternative splicing patterns. In the heart, a number of alternative splicing events occur between postnatal days 1 and 28 as described by Giudice *et al.* (46). RNA-Seq datasets from this study were aligned with STAR and the junction output files were imported into Ularcirc. Ularcirc was clearly capable of visualising the alternative splicing patterns between postnatal day 1 and adult tissue (Supplementary Figure S5) and we therefore concluded that the FSJ output files are a valuable resource in determining exon usage patterns of parental transcripts.

The visualization and quantitation of both FSJ and BSJ provide a unique opportunity to cross examine parental transcript expression with circRNA biogenesis. FSJ that span/splice across a BSJ potentially discriminate two previous models proposed for circRNA biogenesis (Supplementary Figure S6). The presence of a FSJ that spans a BSJ would be supporting evidence for lariat derived circRNA biogenesis (Model 1 of (4)) resulting from exon skipping. Conversely the absence of spanning FSJ suggests circRNA biogenesis that is independent of exon skipping (Model 2). From all data sets analysed (Supplementary Table S1) there was a distinct absence of FSJ that span BSJ, even when the proportion of BSJ relative to other FSJ was >10% (Supplementary Figure S7). One interpretation of this result is that the majority of circRNA are actively produced and not by-products of lariats generated during canonical splicing. This was a similar conclusion to the study performed by Aufiero *et al.* (47). However as standard RNA-seq does not reliably capture FSJ from unstable linear transcripts we cannot completely rule out that circRNA biogenesis may also result from aberrant transcription and splicing events as reported in (48).

**Figure 4.**
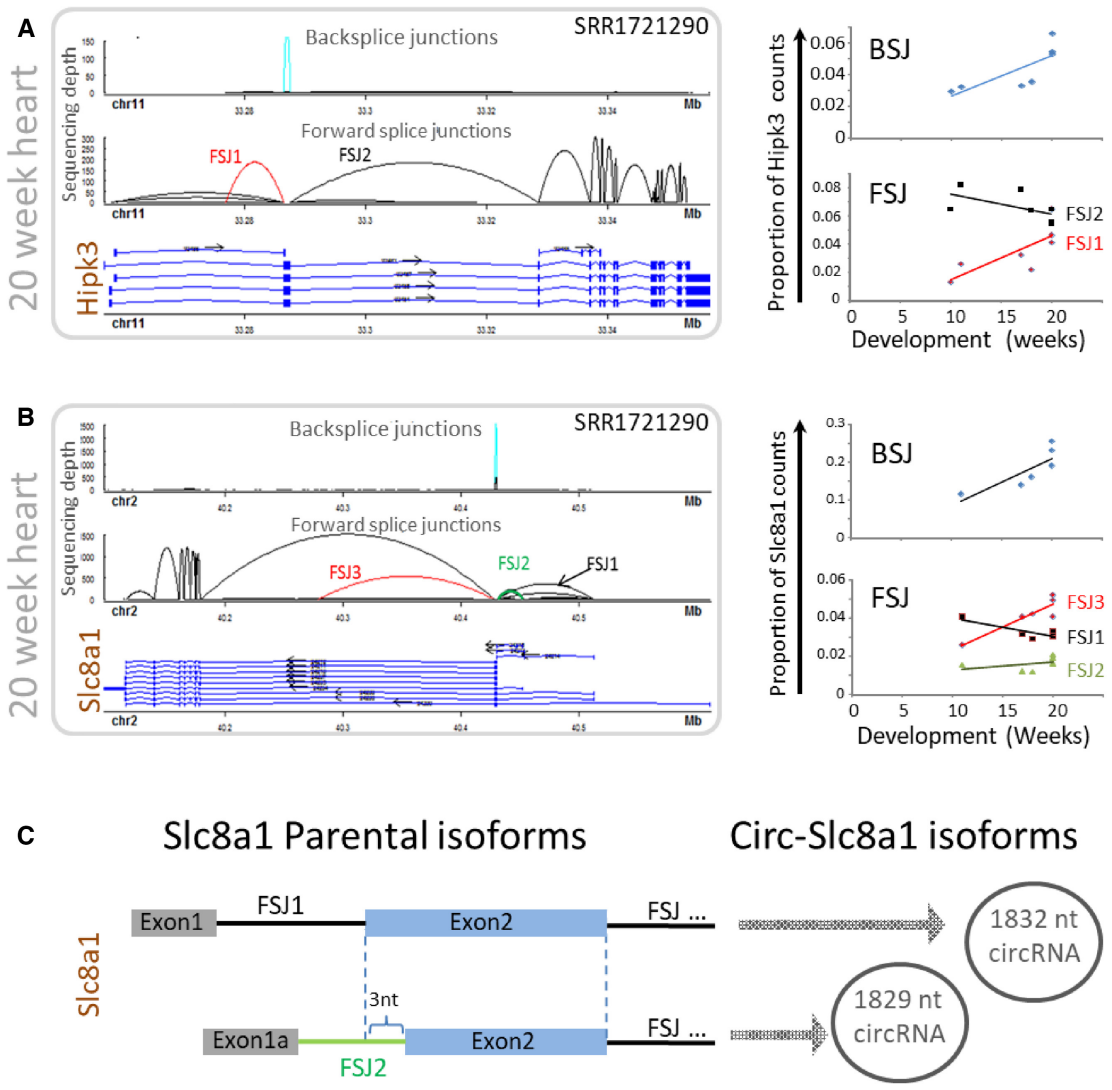
Examples of novel forward splice junction (FSJ) expression across development that is associated with BSJ (circRNA) expression. (**A**) circRNA from HipK3 is derived from exon 2 (light blue) whose expression correlates with a novel canonical FSJ of the parental gene (red). (**B**) Two circRNA from Slc8a1 are derived from exon 2 (previously reported as exon 1) that correlate with different exon expression patterns of the parental transcript. (**C**) Each Slc8a1 circRNA is likely to derive from parental transcript isoforms that utilize different exon 2 splicing acceptor that differ by 3 nt.

We next used Ularcirc to analyse expression patterns of abundant circRNA and FSJ from parental transcripts across embryonic heart development data sets (49). Interestingly we identified two genes that express abundant circRNA, *Hipk3* and *Slc8a1*, whose expression correlated with novel parental canonical FSJ (red splice junctions in Figure 4A and B). For both *Hipk3* and *Slc8a1*, the novel FSJ was either upstream or downstream of the circRNA respectively. The correlation in circRNA and novel FSJ abundance suggests that for these examples, novel transcript isoforms produce the circRNA.

Human *Slc8a1* produces two abundant circRNA isoforms that are upregulated throughout heart development. Previous analysis of *Slc8a1* circRNA concluded that both circRNA isoforms are derived from alternative processing of exon 1 (49), which we thought may be unlikely. We therefore examined different gene models for *Slc8a1*. Refseq (13 July 2017 annotation release) annotates five Slc8a1 transcript isoforms of which only one contains an exon positioned upstream of the circularized exon. Genecode (v24) annotates eight Slc8a1 transcript isoforms of which four have an exon positioned upstream of the circularized exon (Figure 4B). Ularcirc visualization identified that heart *Slc8a1* linear transcripts isoforms incorporate these alternative upstream exons. Therefore *Slc8a1*-circRNA should be annotated as deriving from exon 2. Furthermore, two of the dominant alternative upstream exons utilize distinct acceptor sites within exon 2 (FSJ1 and FSJ2 in Figure 4B). These different acceptor sites match perfectly to the BSJ of the two Slc8a1 circRNA isoforms. FSJ2 aligns to minor circRNA and FSJ1 aligns to major circRNA isoform (Figure 4C). Interestingly the novel linear and circRNA Slc8a1 transcript isoforms are not evolutionary conserved as the analysis of a mouse ventricle time course data set only identified one circRNA species that correlates with a single transcript isoform (Supplementary Figure S8). However, mouse Slc8a1 circRNA are also derived from exon 2 supporting the notion that Slc8a1 circRNA should be annotated as being processed from exon2 of the parental transcript isoforms.

### Internal circRNA splicing

**Figure 5.**
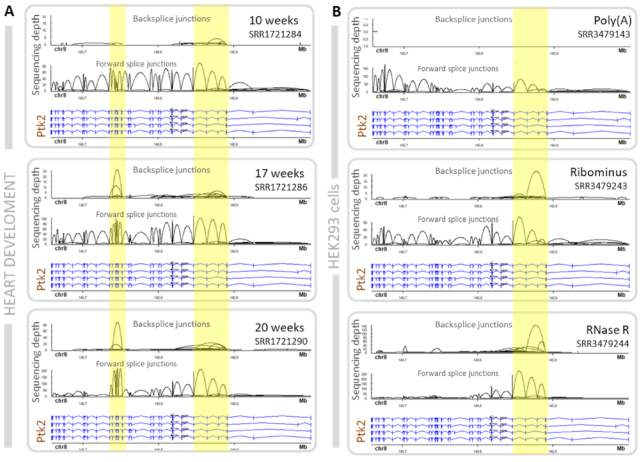
Ptk2 circRNA in either human heart and Hek293 cells. (**A**) Heart PTK2 circRNA are mainly derived from the 3′ exons of parental transcript (Note PTK2 is encoded on negative strand). Expression levels of FSJ (highlighted in yellow) within the boundaries of BSJs increase across development suggesting internal circRNA splicing. (**B**) PTK2 circRNA are mainly derived from the 5′ exons of parental transcript expressed in the HEK293 cell line and are detected in Ribominus and RNase-R libraries. FSJ within boundaries of BSJ (highlighted in yellow) are significantly enriched after RNase R treatment, suggesting internal circRNA splicing.

CircRNA are typically derived from one or more internal exons of parental transcripts. Deciphering internal exon composition typically involves specialized algorithms and/or long-read sequencing analysis (29). We identified two scenarios where Ularcirc can also provide insights into the internal splicing patterns of circRNA, without the need for long-read sequencing. The first scenario involves looking for upregulated FSJ (FSJ hotspot), that resides within the coordinates of an abundant BSJ, relative to the other FSJ of the parental transcript. Ideally, the expression changes of an FSJ hotspot should correlate with the expression changes of the BSJ. Ribominus developmental time course data sets are a great resource for this type of analysis, particularly for developmentally upregulated circRNA. One such example is circRNA derived across five central exons of PTK2 in the developing heart. The increase of FSJ within the BSJ boundaries are following the increase of BSJ while the FSJ outside the boundaries are not, suggesting that they are derived from PTK2 circRNA (Figure 5A). Total sequence coverage across PTK2 confirmed that these circRNA exons were more abundant than other exons (Supplementary Figure S9A). We quantitated circRNA FSJ of the most abundant PTK2 circRNA using CIRI-FULL and confirmed an increasing abundance of internalized splice junctions (Supplementary Figure S9B and C).

The second scenario also involves identifying FSJ hotspots that correlate with BSJ expression but utilizes data sets generated from RNase R treated RNA. We examined matched poly(A), ribominus, and RNase R public data sets generated from the HEK293 human cell line. As expected looking at *PTK2*, the circRNA isoforms detected by the BSJ in the ribominus data are enriched in the RNase R dataset but are absent, as visualized by the lack of BSJ, in the poly(A) data. Concomitantly, the FSJ within BSJ boundaries are lower in the poly(A) dataset compared to the ribominus dataset while counts of all FSJ junctions across the length of the rest of the gene are similar between the two datasets. Reciprocally, the only FSJ detected in the RNase R data are within the boundaries of the BSJ, which again supports that the exons within FSJ boundaries enter the composition of *PTK2* circRNA (Figure 5B). We believe algorithms could be developed to take advantage of this trend and extrapolate internal splice junction composition of circRNA. Furthermore, these two examples of circRNA (Figure 5A and B) also reveal that different tissues are capable of producing different circRNA isoforms from the same gene. CircRNA derived from the 3′ end of PTK2 are predominantly processed in Hs68 fibroblast cells, while circRNA derived from the 5′ end are predominantly processed in heart tissue. This suggests that certain tissue-specific factors can drive circRNA biogenesis.

### Complex circRNA formation

**Figure 6.**
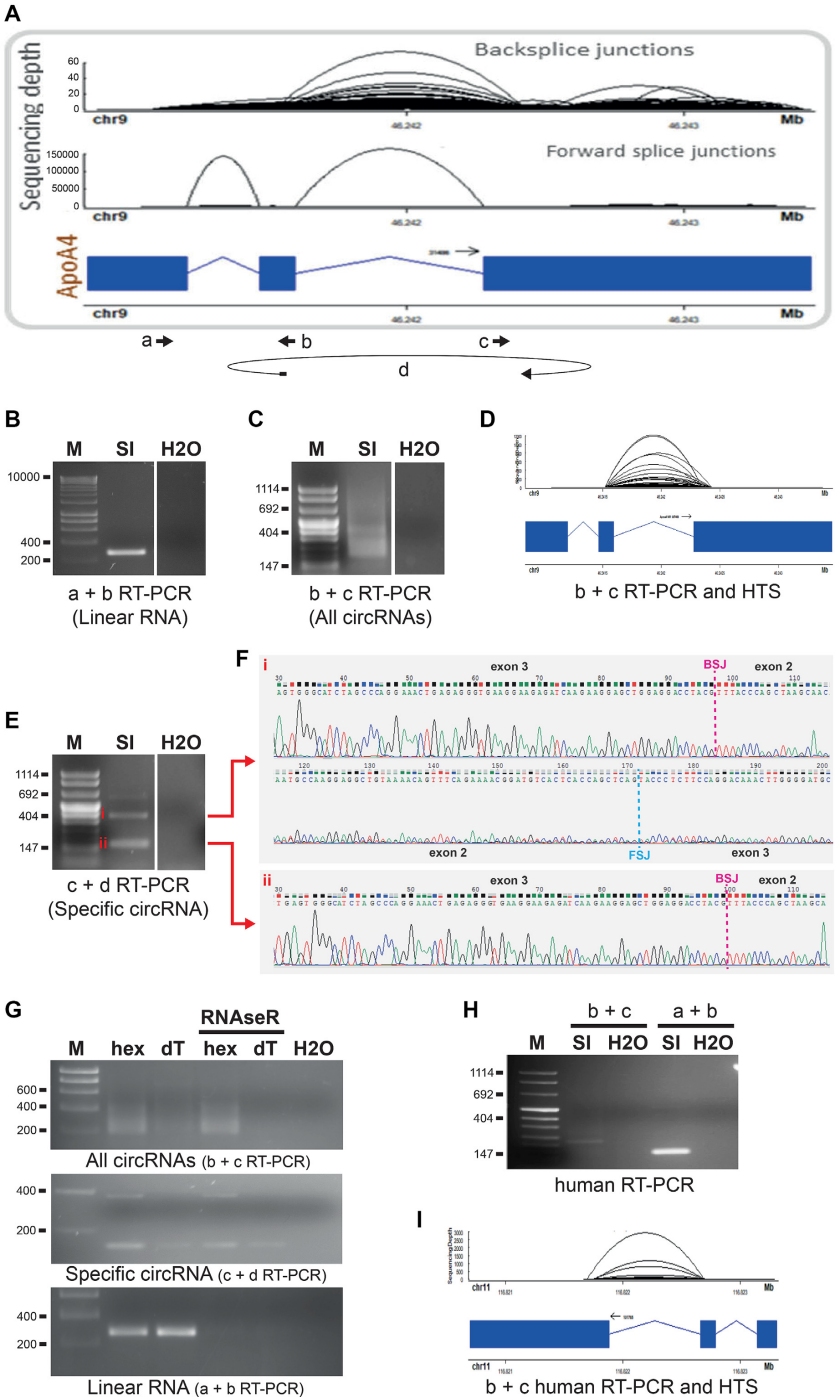
Complex circRNA formation from the *ApoA4* gene. (**A**) High throughput sequencing and Ularcirc analysis of total RNA extracted from epithelial cells of the murine small intestine (SI) identified many BSJs derived from multiple positions within exons 2 and 3 of the *ApoA4* gene, as visualized with Ularcirc. Bold arrows indicate the position and the orientation of the primers used for PCR amplifications parts B, C, D, E, G, H and I. Primer d overlaps a specific BSJ. (**B**) Agarose gel visualization of the parental (linear) ApoA4 transcript amplified by RT-PCR from total RNA with convergent primer a and b. (**C**) Visualization of multiple backsplice transcripts (smear) amplified with divergent primer b and c. (**D**) High throughput sequencing (HTS) of the products amplified with divergent primers b and c captures the same range of BSJs as seen in part A. (**E**) Visualization of a range of concatenated circRNA products (i.e. rolling circle amplification) amplified with divergent primers c and d (d overlapping a specific BSJ). (**F**) Sanger sequencing of RT-PCR products (i) and (ii) isolated from the agarose gel part E. Positions of the BSJ and the FSJ are indicated. (**G**) Same RT-PCR performed part B, C and E but from total RNA treated or not with RNAseR and reverse transcribed with random hexamers (hex) or oligo(dT)_20_ (dT). (**H**) Visualization of the backsplice and the parental (linear) *APOA4* transcripts amplified with divergent primers b and c and convergent primers a and b respectively, from human small intestine RNA. (**I**) High throughput sequencing of the products amplified with divergent primers b and c from human small intestine RNA. H2O, water-only control; M, marker (size, i.e. bp, indicated on the left of the gels).

Ularcirc can also capture the formation of more complex circRNA. Using a dataset generated in-house from murine epithelial cells of the small intestine, we identified an extreme example of complex circRNA formation derived from *ApoA4. ApoA4* encodes the secreted Apolipoprotein A4 which is important for lipid metabolism (50). These circRNA are not processed from defined linear acceptor/donor splice sites but rather a more disorganized spread of positions located within exonic regions (Figure 6A). These circRNA have RAD scores that are close to 0.5, but the FSJ support values are 0. We reasoned that if these BSJ represented genuine circRNA, the majority of the BSJ could be validated in a PCR reaction using a single pair of divergent primers. Indeed, PCR amplification with this pair of primers produced a range of products that was represented by a smear on an agarose gel with a spread of larger incremental fragments (Figure 6B and C). The PCR product was then prepared as a sequencing library using fusion primers (see material and methods) and the resulting reads reproduced what we identified from the original RNA-Seq data (Figure 6D).

To try to specifically single-out one type of circRNA, we replaced one of the primers of the divergent pair with a primer that overlaps one defined BSJ. The PCR amplified an expected dominant ∼200nt amplicon as well as a series of larger but less represented amplicons spaced at ∼200nt intervals (Figure 6E). We suspected these larger amplicons were concatemers of the primary amplicon and very likely derived from rolling circle amplification from the reverse transcriptase from the circularized RNA (51). Sanger sequencing of the cloned fragments extracted from the gel confirmed this to be the case (Figure 6F). To further prove circularization, we reasoned that circRNA should be preferentially amplified by PCR from random hexamer generated cDNA than oligo(dT) generated cDNA, as circRNA do not have a poly(A) tail. Indeed, all ApoA4 divergent primer pairs preferentially amplified products from random hexamer cDNA compared to oligo(dT) cDNA (Figure 6G, top and middle gels). No such difference was seen for control convergent primer pairs designed to amplified linear ApoA4 transcript amplicons (Figure 6G, bottom gel). In addition, we showed that the products obtained with convergent primers, but not the products obtained with divergent primers, were sensitive to treatment with RNAseR which specifically hydrolyses linear RNAs but not circRNA (Figure 6G). Finally, we demonstrated that *ApoA4* RNA circularization is conserved in human by performing *ApoA4* divergent RT-PCR using RNA sample from human small intestine (Figure 6H, I). Altogether, these results support the formation of non-canonical ApoA4 circRNA.

Analysis of the top 200 ApoA4 BSJ revealed the lack of canonical acceptor and donor splice sites (Supplementary Table S3), which emphasizes the complexity of circRNA formation. We speculate that RNA binding proteins that are not known to be classical splicing factors must be involved in this process.

### Ularcirc complements and completes existing gene models

Ularcirc inbuilt circRNA detection method assembles backsplice junction counts independently of existing gene models. Both the RAD score and the FSJ support score metrics are also assembled without the need of existing gene models. Therefore, Ularcirc is suited for circRNA discovery in both model and non-model organisms. When gene models are available Ularcirc provides functionality to assign gene names to identified BSJ.

**Figure 7.**
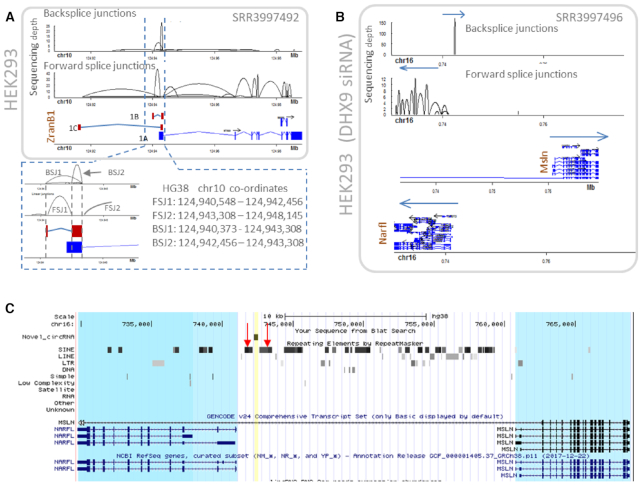
Novel abundant circRNA that do not align to current gene annotations. (**A**) We used Ularcirc to identify that ZranB1 has two additional alternative first exons which are annotated as 1B and 1C, which are both expressed in HEK293 cells. The pre-existing annotation is labelled as 1A. Two circRNA isoforms are generated from transcript isoforms 1B and 1C as the coordinates of these backsplice junctions match perfectly to the novel transcript isoforms (refer zoomed inset). (**B**) The most abundant circRNA from *DHX9* knockdown cells was identified using Ularcirc and does not align to any canonical annotated or expressed exon. Two neighboring genes that are transcribed from different strands are *NARFL* and *MSLN*. The novel circRNA is transcribed from the same strand as *MSLN*, but from an intronic region. (**C**) UCSC browser screenshot highlighting the proximity of ALU repeat elements (red arrows) that surround the novel circRNA (highlighted in yellow). Neighbouring genes are highlighted in blue.

We decided to compare Ularcirc inbuilt circRNA detection to a gene model dependent pipeline. We chose circExplorer2 as both pipelines can use the same chimeric output from the STAR aligner. For this reason, it was not surprising that both detected a similar number of true positives (Supplementary File 1) and reported an identical count for a large portion of reported BSJs (Supplementary Figure S10A). Nevertheless, circExplorer2 may still miss to identify some circRNA produced from genes whose annotations are incomplete. With that in mind, we re-analysed circularization in a data set where DHX9 is knockdown (48), which results in aberrant splicing of linear transcripts and the upregulation of many circRNA. We discovered abundant circRNA that were identified by Ularcirc (after RAD score and FSJ support score filtering) but not by circExplorer2 (Supplementary Figure S10A). As expected, most of these circRNA were derived from exon boundaries not defined in Gencode (release v24). For example, two circRNA were identified to be processed from the 5′ end of Zranb1. Both ZranB1 circRNA utilized the splice donor from exon1 but create a BSJ either within the middle of exon 1 or ∼3kb upstream of exon 1 donor splice junction (Figure 7A). The analysis of FSJ expression patterns from the sequencing data identified that ZranB1 has two alternative start sites that are not currently annotated, which we name 1B and 1C (pre-existing exon 1 now referred to as 1A). Furthermore, exon 1B and 1C splice into exon 1A to a splice acceptor position that exists within the current defined boundaries of 1A. The internal splice site within exon 1A matches the position of one circRNA (Figure 7A inset).

Ularcirc also identified an abundant BSJ that was annotated to be derived from a sub-intronic region of MSLN (Figure 7B). Interestingly, this circRNA was only expressed when *DHX9* is knockdown, suggesting that the circularization process is dependent on an RNA secondary structure recognized by DHX9. Analysis of the surrounding genomic landscape reveals an enrichment of SINE/ALU sequences that flank each side of the circularized sequence (Figure 7C), suggesting these sequences may contribute to circRNA biogenesis. The lack of associated FSJ and linear reads makes it difficult to identify the ‘parental’ transcript. The two most likely possibilities are that the circRNA is derived from an antisense transcript from the NARFL promoter or from the spliced intronic sequence of the MSLN gene. Together these examples demonstrate that gene repositories are still incomplete even for model organisms, and that a comprehensive identification of circRNA, BSJ and FSJ absolutely require a software independent of existing gene models such as Ularcirc.

## DISCUSSION AND CONCLUSION

In this study, we have developed a bioinformatic software package dedicated to circRNA detection, visualization and analysis. Ularcirc is built on the shiny - R framework and utilizes splicing junctions reported by the STAR aligner, as well as circRNA output from CIRI2 and circExplorer2, two validated circRNA detection programs. We designed Ularcirc to follow a systematic workflow involving the generation of tabulated BSJ count tables, integrated genomic visualizations and functional analysis of ORFs and miRNA binding sites. The graphical user interface offers investigators with minimal working knowledge in Bioinformatics the capability to explore the data sets. Furthermore, the integration of bioconductor databases allows the configuration of Ularcirc to render it compatible with the analysis of sequencing data of a wide range of organisms.

To date, the primary goal of existing circRNA software has focused on the performance of detecting circRNA by identifying BSJ which is difficult given the low frequency of BSJ in sequencing data sets. The primary output of these command-line based software are tab delimited tables, generally one per sample, which then requires further examination typically using custom scripts. Ularcirc provides an efficient solution for this step by assembling multiple CIRI2 and circExplorer2 outputs or, alternatively, has an inbuilt method to identify and discover circRNA from STAR chimeric outputs in the absence of a gene model. The inbuilt circRNA method of Ularcirc is not as efficient in filtering out false positive as other dedicated BSJ detection software but it is more capable of discovering abundant novel candidates that do not fit existing gene models. The benefit of Ularcirc is that all assembled BSJ count tables from all compatible sources can be imported, normalized and assessed via PCA, heatmap, and count distribution plots. In this way users can compare and cross reference the output of different methods to maximize discovery.

Currently, there is a ‘lack-of/weakness-in’ the means to normalized BSJ counts. The default approach most studies utilize is to normalize against total BSJ counts. However, given the wide discrepancies in circRNA discovery between software, the accuracy of this method varies. Another approach is to normalize against the total linear reads (spliced reads per billion mapping = SRPBM) (4) or a pre-defined set of canonical genes (18). Ularcirc provides two normalization options, the first is to normalize against total BSJ counts and the second is to normalize BSJ to total gene counts which is analogous to the SRPBM method (4). While evaluation of the best normalization method is out of the scope of the present study, this can now be explored by using the normalization features of Ularcirc.

Another key feature of Ularcirc is the integrated visualization of both FSJ with BSJ. This feature was developed because both FSJ and BSJ are intrinsically linked as both compete and utilize the same splicing machinery components and are assembled using the same junctions (19,20,52). We demonstrated that FSJ analysis can identify novel insights into parental transcript isoforms of circRNA. Importantly these observations would not have been realized using existing gene models. FSJ analysis can provide insights into circRNA biogenesis and one hypothesis for circRNA biogenesis is that circRNA are by-products of exon skipping (4). If circRNA are indeed by products of exon skipping, we would expect to visualize the FSJ that span from the 5′ upstream exon through to the downstream 3′ exon. These splice junctions should be more apparent at the onset of circRNA biogenesis. However, we rarely detected alternative splicing events that could produce circRNA by products (data not shown). This observation agrees with a recent study identifying that most cardiac circRNA arise from constitutive exons and not alternative splicing events (47). However, there are situations where circRNA can accumulate disproportionally to their parental transcript (17,18). These situations most likely arise due to the stable structure of the circRNA and/or active biogenesis by specific RNA binding proteins (18,20,48).

Active circRNA biogenesis raises questions relative to their biological function. We incorporated into Ularcirc two basic functional analysis tools, ORF and miRNA-motif analysis, that enable the screening for potential circRNA function. Implementation of these two features were selected due to previous discoveries associating translational activity and miRNA targeting functions with circRNA (24,25,53,54). Interestingly, we were surprised to identify some circRNA that have an ORF longer than the coding potential of an equivalent linear transcript. Hipk3 circRNA is particularly intriguing as it is present in the cytoplasm of many cell types and has been identified to act as a miRNA sponge (22). Silencing of Hipk3 circRNA inhibits cell growth which has been attributed to miRNA sponge activity. However, with recent studies supporting the ability of circRNA to be translated, Hipk3 circRNA function could also be explained by the translation of a novel isoform from its super-ORF. Under this scenario, the miRNA binding capabilities would be a translational regulatory event rather than pure sponge activity.

As the function of a circRNA is principally deduced from its sequence (e.g. identifying miRNA binding motifs), it has become more and more important to precisely identify internal circRNA splicing for accurate sequence reconstruction. This should be contributed by the development of new sequencing technologies allowing to easily sequence longer reads with high efficicency and high reproducibility. In the meantime, two recent studies have specifically identified circRNA internal splicing patterns through the development of scripts that scan through aligned reads and identify library fragment that contain both a BSJ and a FSJ (29,55). Both methods are constrained to detect FSJ within the sequenced fragment length and therefore can only partially assess the internal splicing patterns of larger circRNA. We demonstrated that Ularcirc integrated visualization can be used to indirectly assess all FSJ within the boundaries of a BSJ internal splicing. From these observations we believe that new approaches can be developed, particularly taking advantage of RNase-R and time-course data sets, to predict the complete internal splicing of circRNA.

Our study has highlighted the efficacy of integrating the analysis of FSJ and BSJ to analyse circRNA. Ularcirc is specifically developed for this task for researchers with minimal bioinformatic skill to perform complex analysis by incorporating integrated visualization combined with a user-friendly graphical user interface. Visualizing complex data sets enables faster processing and appreciation of data trends than scrutinizing data in tabulated formats and reports. Our integrative analysis of FSJ and BSJ using Ularcirc has already gleaned new insights into circRNA biology. We believe that Ularcirc is a valuable technical resource that will significantly enhance the analytical capacity for circRNA research.

## DATA AVAILABILITY

Ularcirc software is available at https://github.com/VCCRI/Ularcirc, which is under GNU general public license v3.0. The public RNA-Seq datasets used in this study are listed in Supplementary Table S1. The generated RNA-seq data setsare available through SRA (PRJNA471490).

Operating system: Platform independent

Programming language: R

Other requirements: See https://github.com/VCCRI/Ularcirc

## Supplementary Material

gkz718_Supplemental_Files

## Notes

Present address: Nicolas Fossat, Copenhagen Hepatitis C Program (CO-HEP), Department of Immunology and Microbiology, University of Copenhagen, and Department of Infectious Diseases, Hvidovre Hospital, Denmark.
