# Supplementary material for: Ularcirc: visualization and enhanced analysis of circular RNAs via back and canonical forward splicing
Source: Nucleic Acids Res. 2019 Aug 22;47(20):e123. doi: 10.1093/nar/gkz718 (PMC6846653; doi:10.1093/nar/gkz718)
Supplement: gkz718_Supplemental_Files [file gkz718_supplemental_files.zip › Supplementary ULarcirc Figures -portrait.June2019.pptx]

## Slide 1
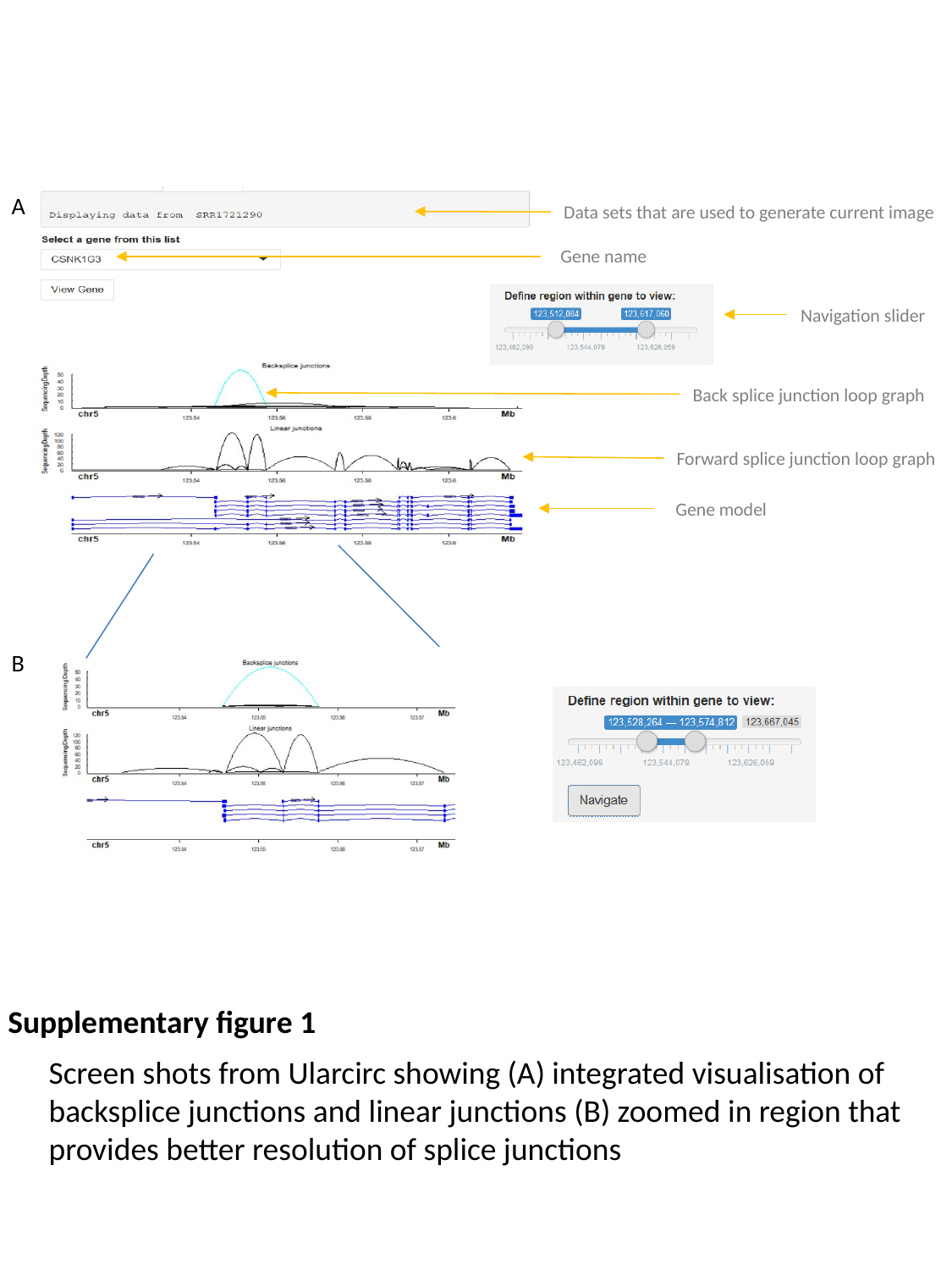

A
B
Data sets that are used to generate current image
Gene name
Navigation slider
Back splice junction loop graph
Forward splice junction loop graph
Gene model
Supplementary figure 1
Screen shots from Ularcirc showing (A) integrated visualisation of backsplice junctions and linear junctions (B) zoomed in region that provides better resolution of splice junctions

## Slide 2
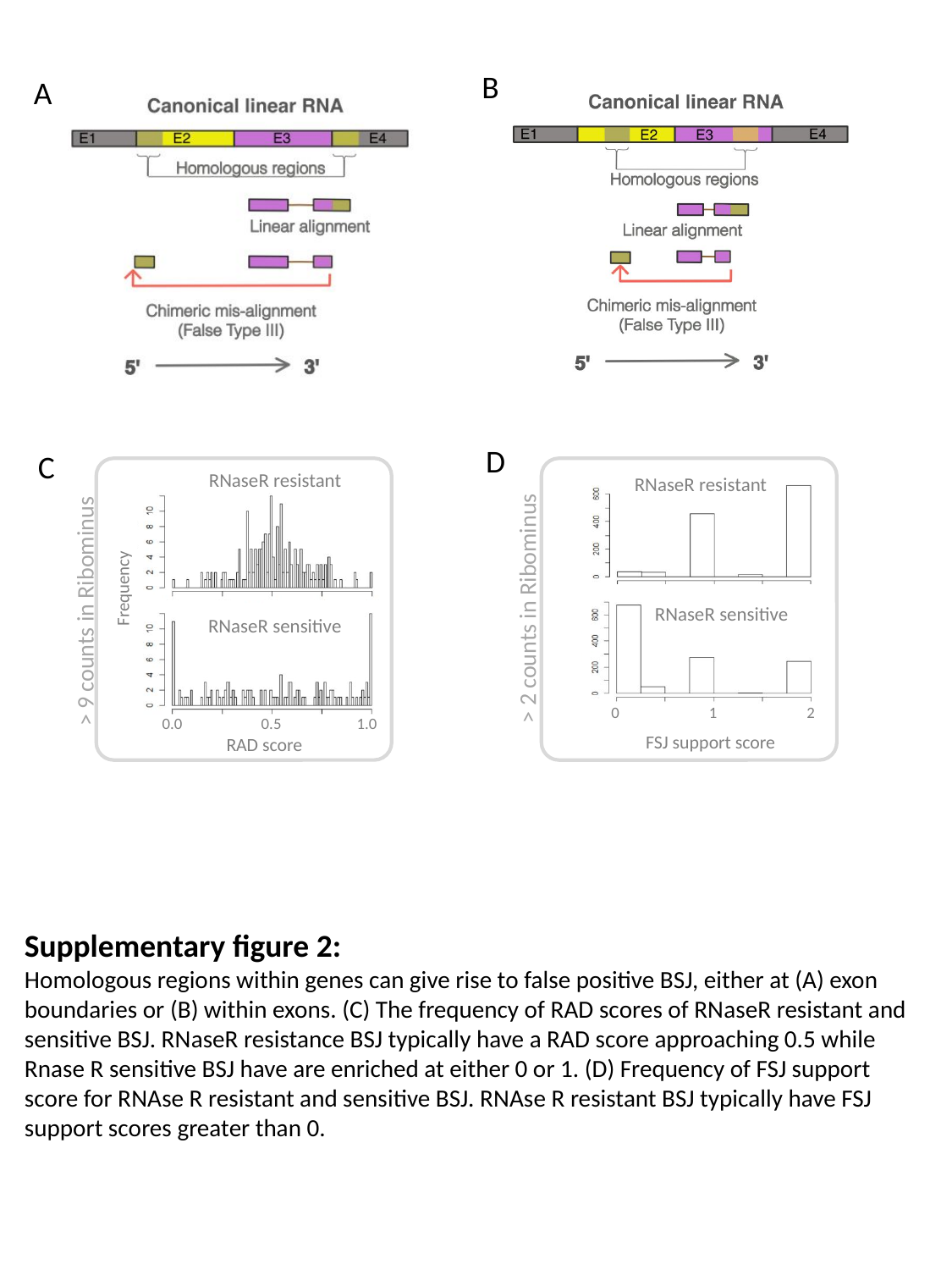

B
A
D
C
RNaseR resistant
RNaseR resistant
0.0 0.5 1.0
RAD score
Frequency
> 2 counts in Ribominus
> 9 counts in Ribominus
RNaseR sensitive
 0 1 2
RNaseR sensitive
FSJ support score
Supplementary figure 2:
Homologous regions within genes can give rise to false positive BSJ, either at (A) exon boundaries or (B) within exons. (C) The frequency of RAD scores of RNaseR resistant and sensitive BSJ. RNaseR resistance BSJ typically have a RAD score approaching 0.5 while Rnase R sensitive BSJ have are enriched at either 0 or 1. (D) Frequency of FSJ support score for RNAse R resistant and sensitive BSJ. RNAse R resistant BSJ typically have FSJ support scores greater than 0.

## Slide 3
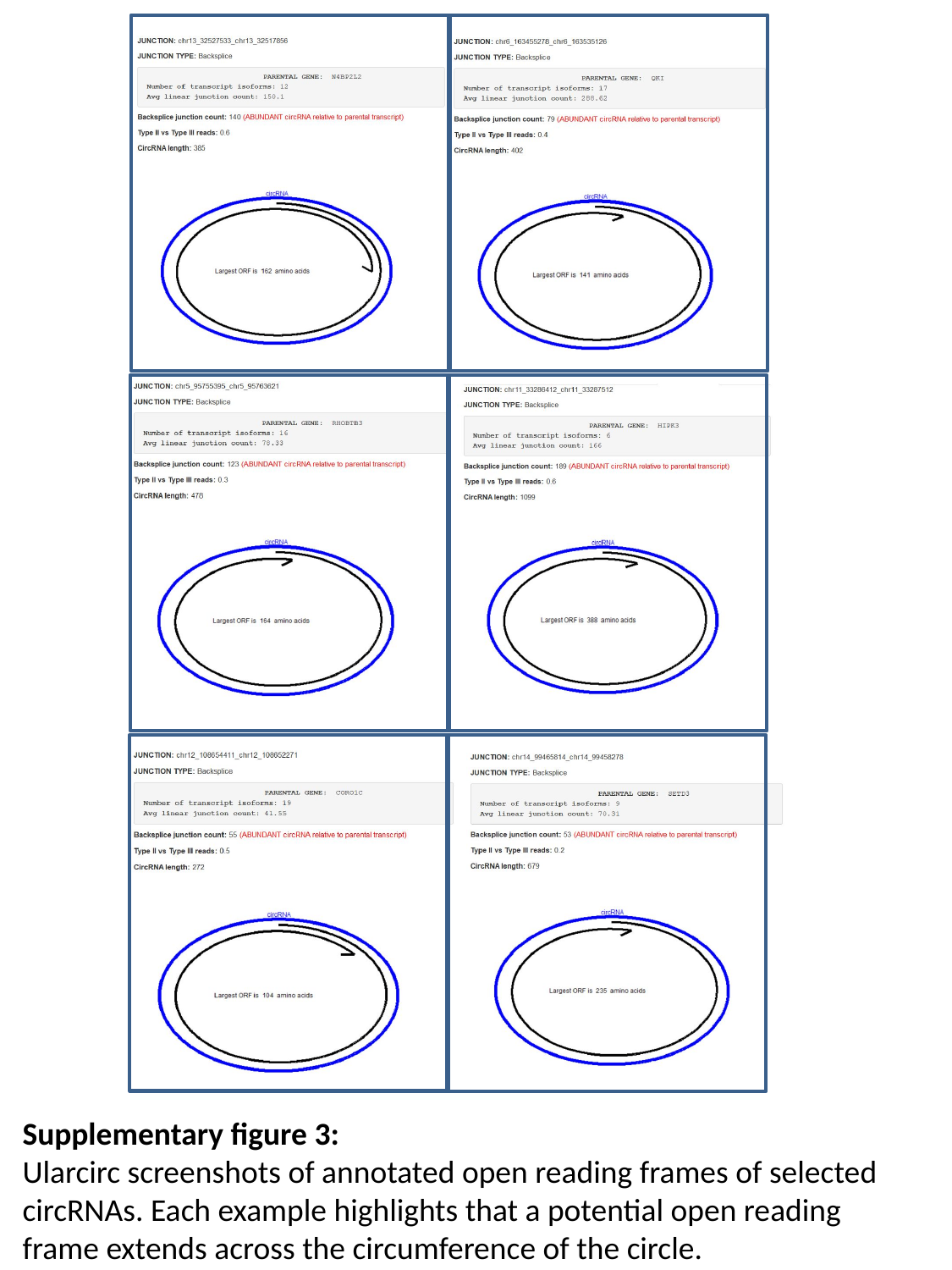

Supplementary figure 3:
Ularcirc screenshots of annotated open reading frames of selected circRNAs. Each example highlights that a potential open reading frame extends across the circumference of the circle.

## Slide 4
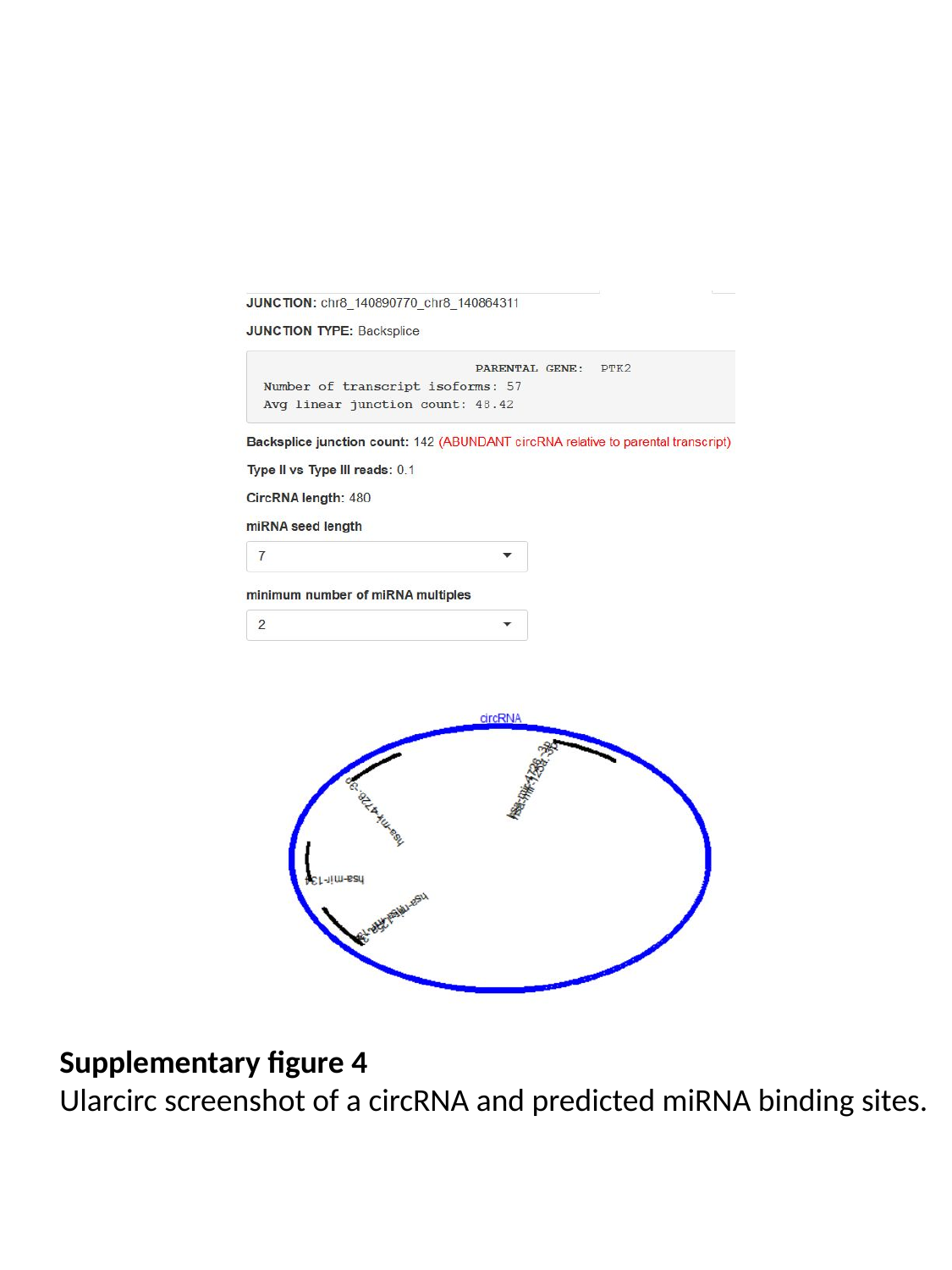

Supplementary figure 4
Ularcirc screenshot of a circRNA and predicted miRNA binding sites.

## Slide 5
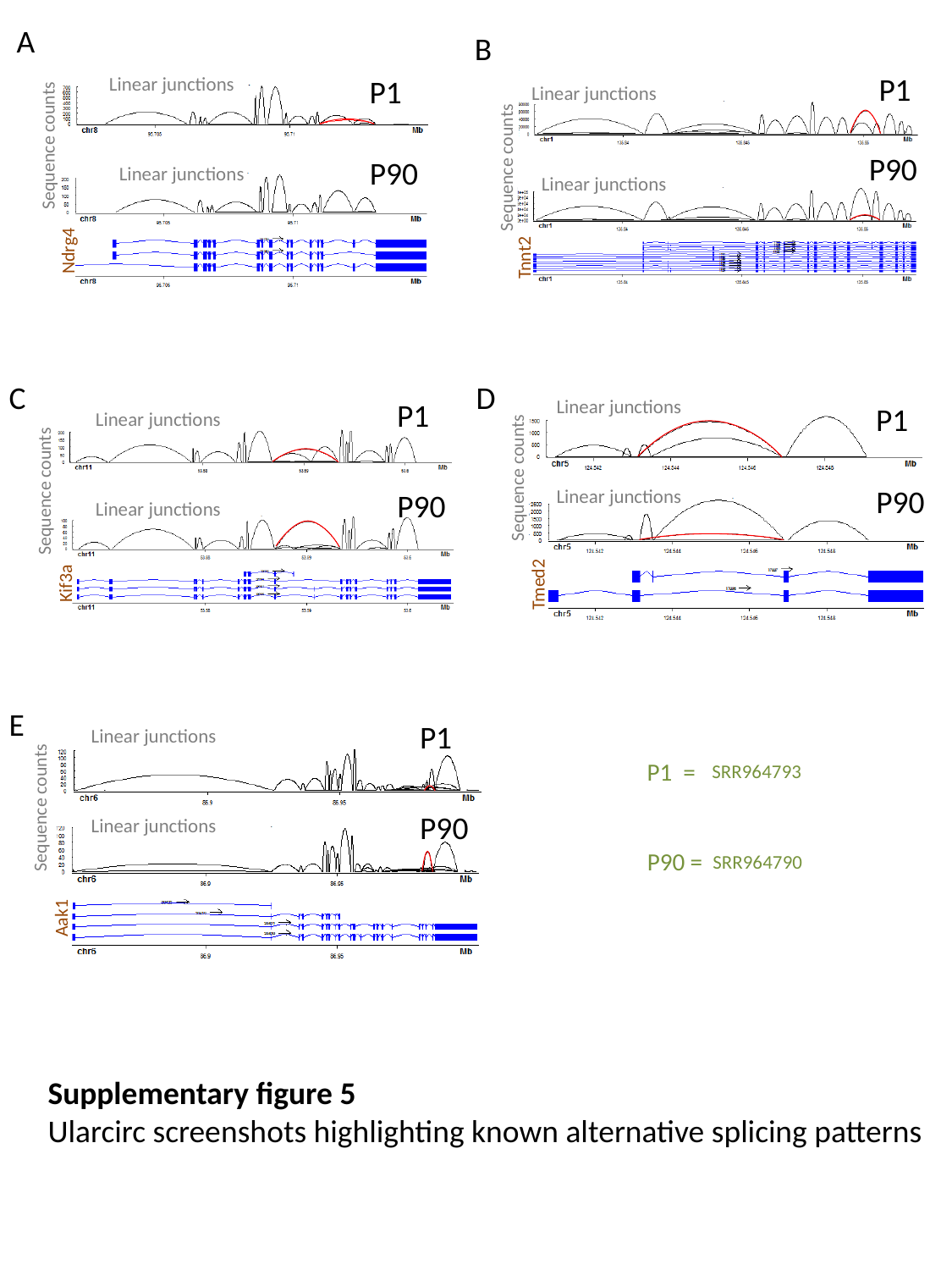

A
B
P1
Linear junctions
P90
Sequence counts
Linear junctions
Tnnt2
Linear junctions
P1
Sequence counts
P90
Linear junctions
Ndrg4
C
D
Linear junctions
P1
Sequence counts
P90
Linear junctions
Tmed2
P1
Linear junctions
Sequence counts
P90
Linear junctions
Kif3a
E
P1
Linear junctions
Sequence counts
P90
Linear junctions
Aak1
P1 =
SRR964793
P90 =
SRR964790
Supplementary figure 5
Ularcirc screenshots highlighting known alternative splicing patterns

## Slide 6
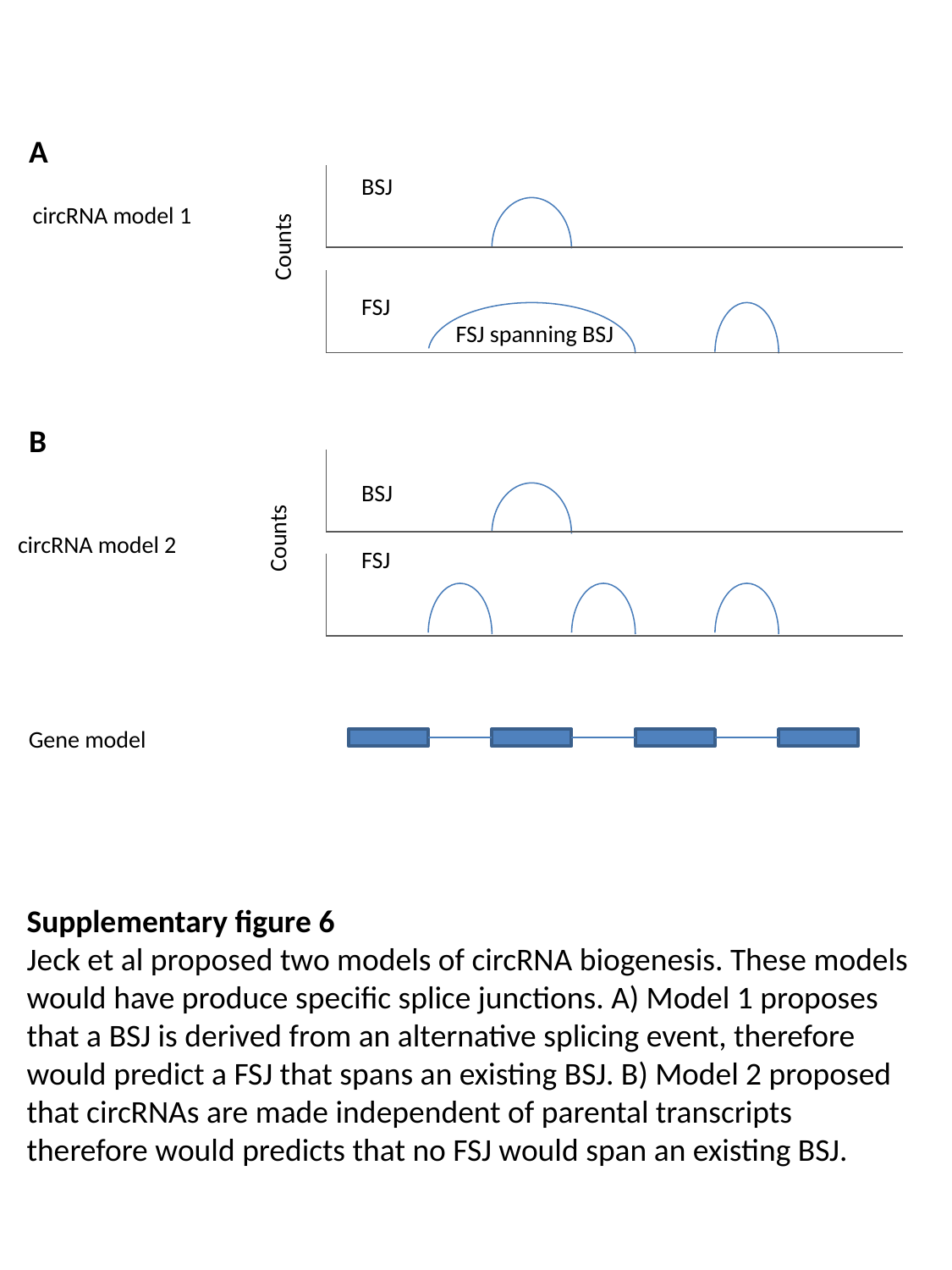

A
BSJ
circRNA model 1
Counts
FSJ
FSJ spanning BSJ
B
BSJ
Counts
circRNA model 2
FSJ
Gene model
Supplementary figure 6
Jeck et al proposed two models of circRNA biogenesis. These models would have produce specific splice junctions. A) Model 1 proposes that a BSJ is derived from an alternative splicing event, therefore would predict a FSJ that spans an existing BSJ. B) Model 2 proposed that circRNAs are made independent of parental transcripts therefore would predicts that no FSJ would span an existing BSJ.

## Slide 7
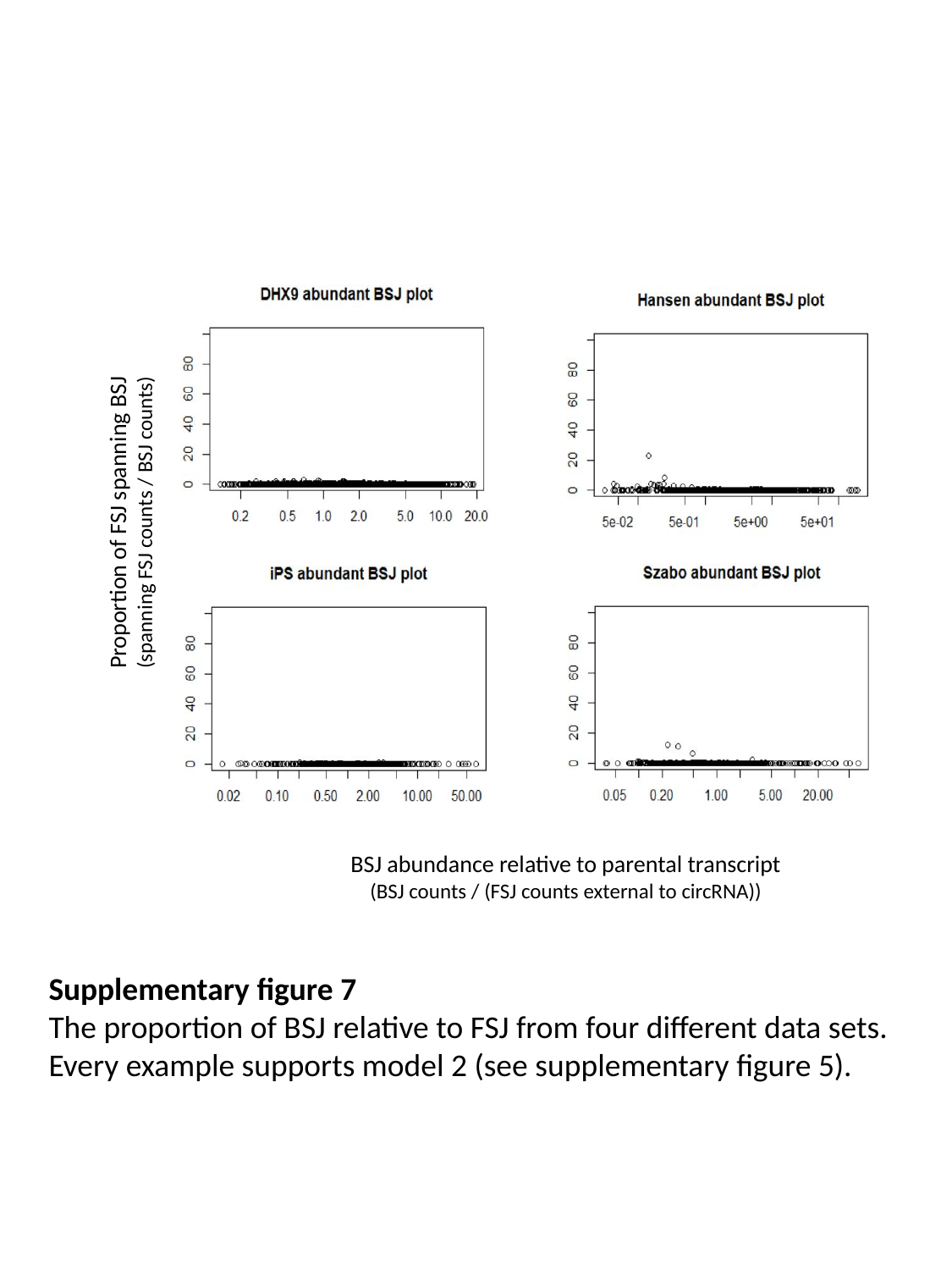

Proportion of FSJ spanning BSJ
(spanning FSJ counts / BSJ counts)
BSJ abundance relative to parental transcript
(BSJ counts / (FSJ counts external to circRNA))
Supplementary figure 7
The proportion of BSJ relative to FSJ from four different data sets. Every example supports model 2 (see supplementary figure 5).

## Slide 8
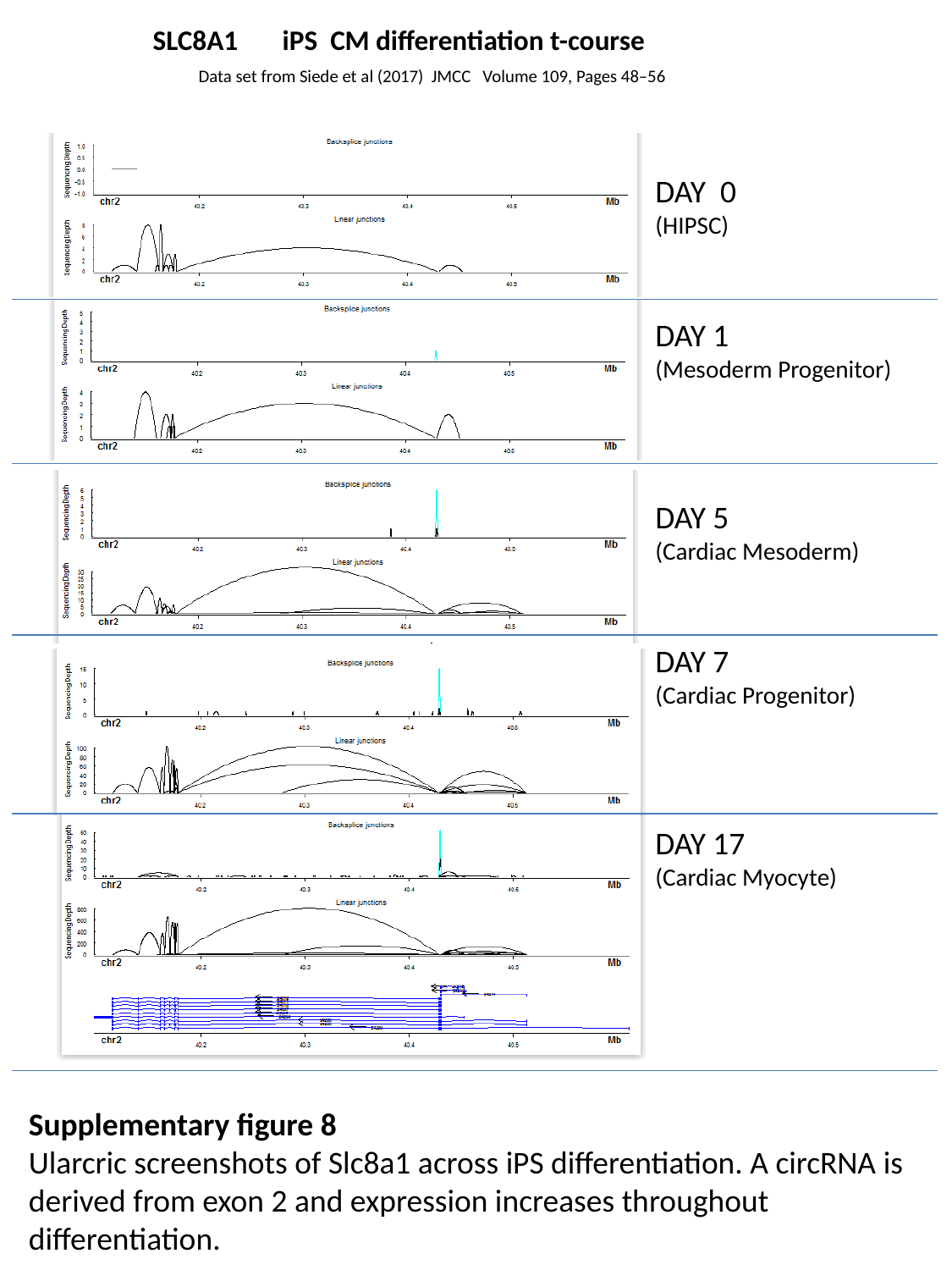

SLC8A1 iPS CM differentiation t-course
Data set from Siede et al (2017) JMCC Volume 109, Pages 48–56
DAY 0
(HIPSC)
DAY 1
(Mesoderm Progenitor)
DAY 5
(Cardiac Mesoderm)
DAY 7
(Cardiac Progenitor)
DAY 17
(Cardiac Myocyte)
Supplementary figure 8
Ularcric screenshots of Slc8a1 across iPS differentiation. A circRNA is derived from exon 2 and expression increases throughout differentiation.

## Slide 9
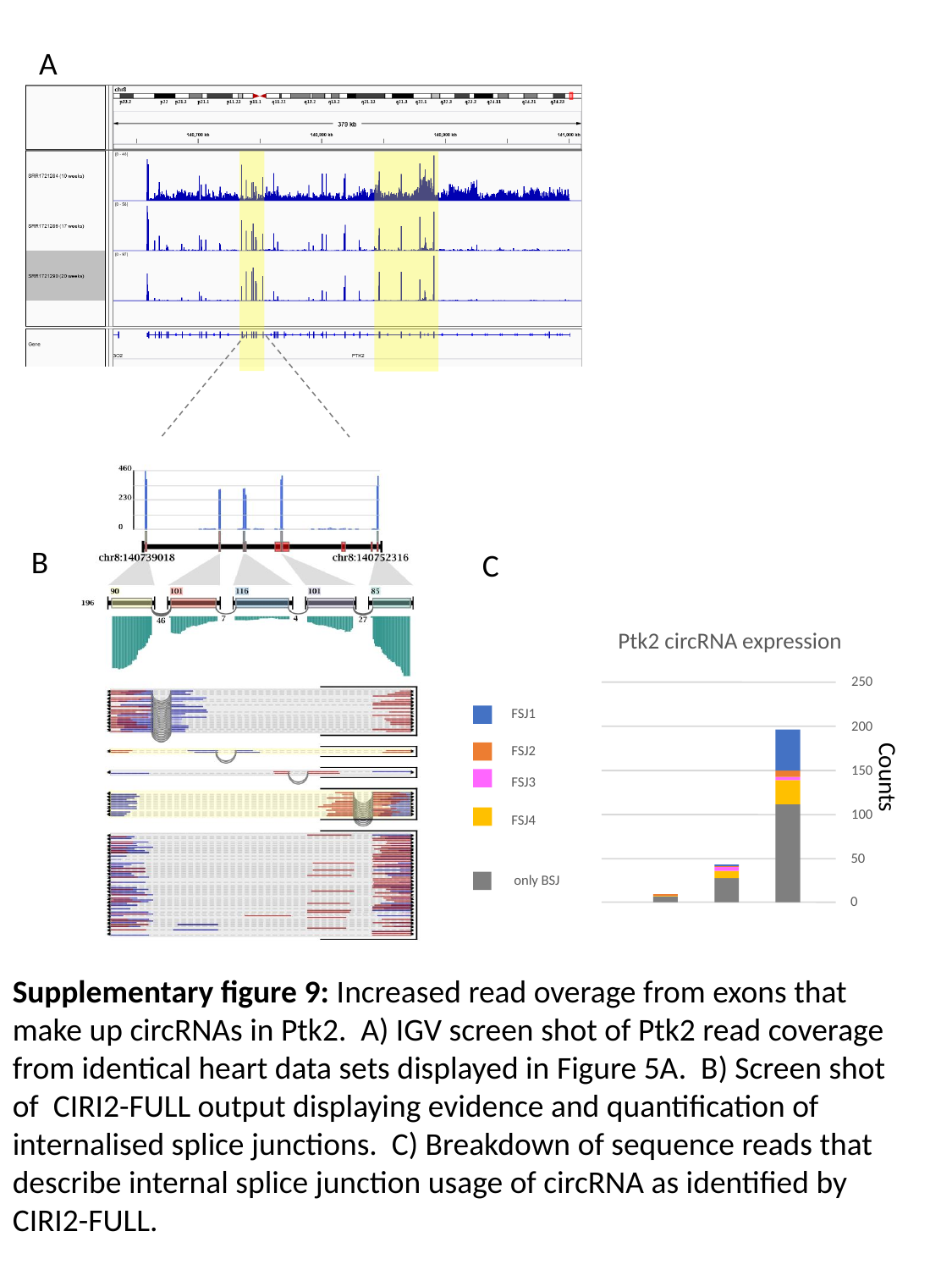

A
B
C
Ptk2 circRNA expression
250
FSJ1
200
FSJ2
Counts
150
FSJ3
100
FSJ4
50
only BSJ
0
Supplementary figure 9: Increased read overage from exons that make up circRNAs in Ptk2. A) IGV screen shot of Ptk2 read coverage from identical heart data sets displayed in Figure 5A. B) Screen shot of CIRI2-FULL output displaying evidence and quantification of internalised splice junctions. C) Breakdown of sequence reads that describe internal splice junction usage of circRNA as identified by CIRI2-FULL.

## Slide 10
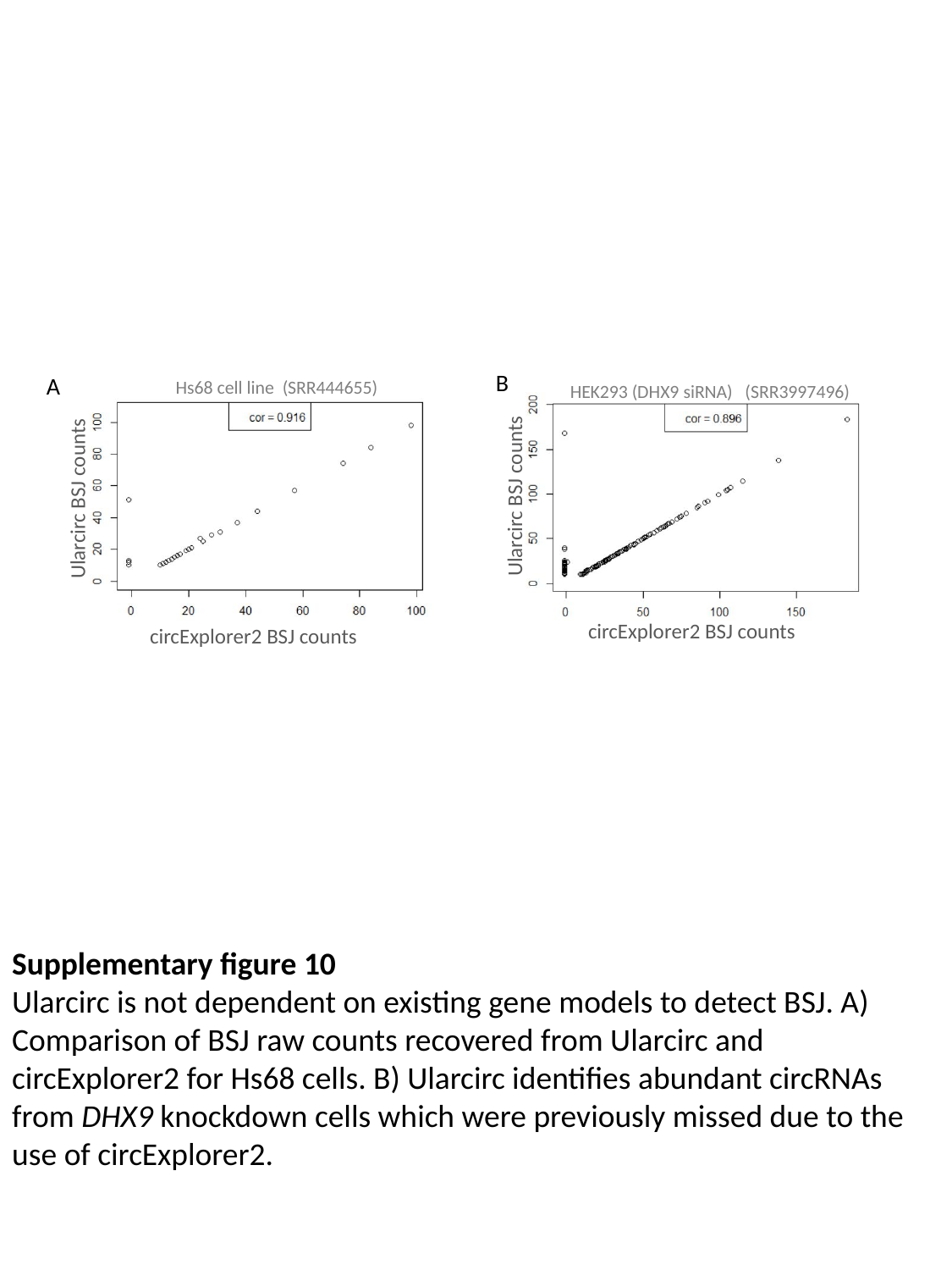

B
A
Hs68 cell line (SRR444655)
HEK293 (DHX9 siRNA) (SRR3997496)
Ularcirc BSJ counts
Ularcirc BSJ counts
circExplorer2 BSJ counts
circExplorer2 BSJ counts
Supplementary figure 10
Ularcirc is not dependent on existing gene models to detect BSJ. A) Comparison of BSJ raw counts recovered from Ularcirc and circExplorer2 for Hs68 cells. B) Ularcirc identifies abundant circRNAs from DHX9 knockdown cells which were previously missed due to the use of circExplorer2.
ASAP1 exon modification
