# Supplementary material for: Ularcirc: visualization and enhanced analysis of circular RNAs via back and canonical forward splicing
Source: Nucleic Acids Res. 2019 Aug 22;47(20):e123. doi: 10.1093/nar/gkz718 (PMC6846653; doi:10.1093/nar/gkz718)
Supplement: gkz718_Supplemental_Files [file gkz718_supplemental_files.zip › Ularcirc Supplementary file1.NAR_resubmission.10thApril2019.docx]

**RAD score ground truth analysis**

We obtained a list of 1834 human circRNA that were previously classified to be true positives due to their resistance to RNaseR degradation [1, 2]. We successfully reconstructed 1832 circRNA sequences using the same method implemented in Ularcirc (refer material and methods of main manuscript). The size range of these reconstructed circRNA ranged from 103 to 3601 (**Figure A**) having a median and mean of 411nt and 511nt respectively.


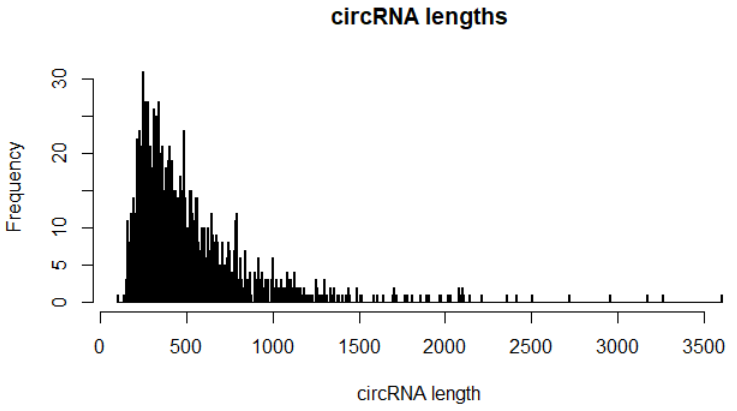


**Figure A** The distribution of read lengths of the 1832 RNaseR resistant circRNA

We generated a fastq data set (fragment size 300bp, read length 100nt) that captured the BSJ of the 1832 true positive circRNA. For each BSJ we generated up to 10 different type II, III and IV paired end fastq reads using the Ularcirc function “BSJ_Fastq_Generate”, generating a total of 53949 read pairs. Each read was encoded with an identification code that enabled quick post alignment cross referencing. Because we definitively know the origins of all reads in this synthetically generated data set we defined it as the ground truth. This data set was used to identify the accuracy in detecting type II and type III reads from chimeric output file CIGAR strings.

The chimeric junction output file generated by the STAR aligner contains two CIGAR strings which represent the donor and acceptor positions of a junction. We reasoned that the total match length encoded in donor and acceptor CIGAR of type II and type III alignments should be different lengths. For more than 99% of read pairs the matched lengths of the donor CIGAR string were larger for type II alignments than type III alignments. Conversely the matched lengths of the acceptor CIGAR string were larger for type III alignments (99.1%). Type II and III alignments that were not identified via these rules were largely derived from small library fragments (i.e. < 190nt) where each read pair captured the BSJ.

**STAR alignment assessment on ground truth data sets**

In 2018, version 2.6 of the STAR aligner was released which incorporated a new improved chimeric read detection algorithm. To measure the improvement in detecting BSJ in this release, we examined the performance of STAR v2.6 and STAR v2.5 in detecting circRNA from the following two synthetically generated fastq ground truth data sets.

1. 100bp paired end reads, library fragment size = 300bp.
2. 100bp paired end reads, library fragment size = 150bp.

From the 1832 reconstructed circRNA data set, we identified 1736 that had a length greater than 200nt. Therefore, for 100bp paired end reads, the majority of the 300bp fragment library would contain non-overlapping read pairs. Conversely, for the 150bp fragment library, every read pair would contain overlapping sequence. In total there were 53949 sequencing reads generated for the 300bp fragment library and 36620 reads generated for the 150bp fragment library.

The 300bp fragment library and 150bp fragment library was aligned with STAR version 2.5 and 2.6. The chimeric output junction file was analysed to determine how many reads were identified to capture a BSJ. From this analysis, we identified that both version of STAR detected a similar proportion (80.5% and 83.5% for v2.5 and v2.6 respectively) of BSJ from the 300bp non-overlapping fragment library (**Figure B**). However, the performance between STAR versions was very different for the 150bp fragment library. STAR v2.6 detected a similar proportion of BSJ from the 150bp as with the 300bp fragment library (78% vs 83.5%), whereas STAR v2.5 only detected 48%.


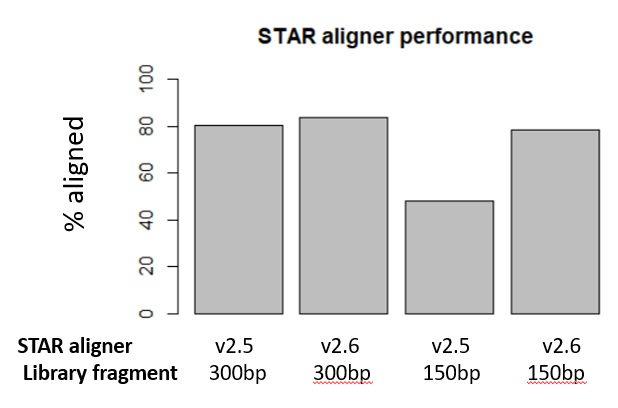


**Figure B:** Performance of different versions of the STAR aligner in detecting BSJ.

Knowing the ground truth allowed the analysis of BSJ reads that were not reported by the STAR aligner. Both STAR aligners failed to detect the majority of BSJ when positioned within the first 20nt of the 5’ end of a read (**Figure C i and ii**). This can be explained from the minimal limit parameter we defined for the STAR aligner to detect chimeric junctions (option “--chimSegmentMin 15”). Interestingly, a large portion of BSJ were detected by either version of the STAR aligner when positioned within the last 20nt of a read (i.e at the 3’ end). Version 2.5 of the STAR aligner also failed to detect BSJ when captured by overlapping read pairs (**Figure C i**), and this issue has been rectified in version 2.6 (**Figure 3B**). We therefore strongly recommend the use of STAR aligner 2.6 for the detection of circRNAs. This is particularly important for the detection of shorter circRNA that will be captured in short library fragments.


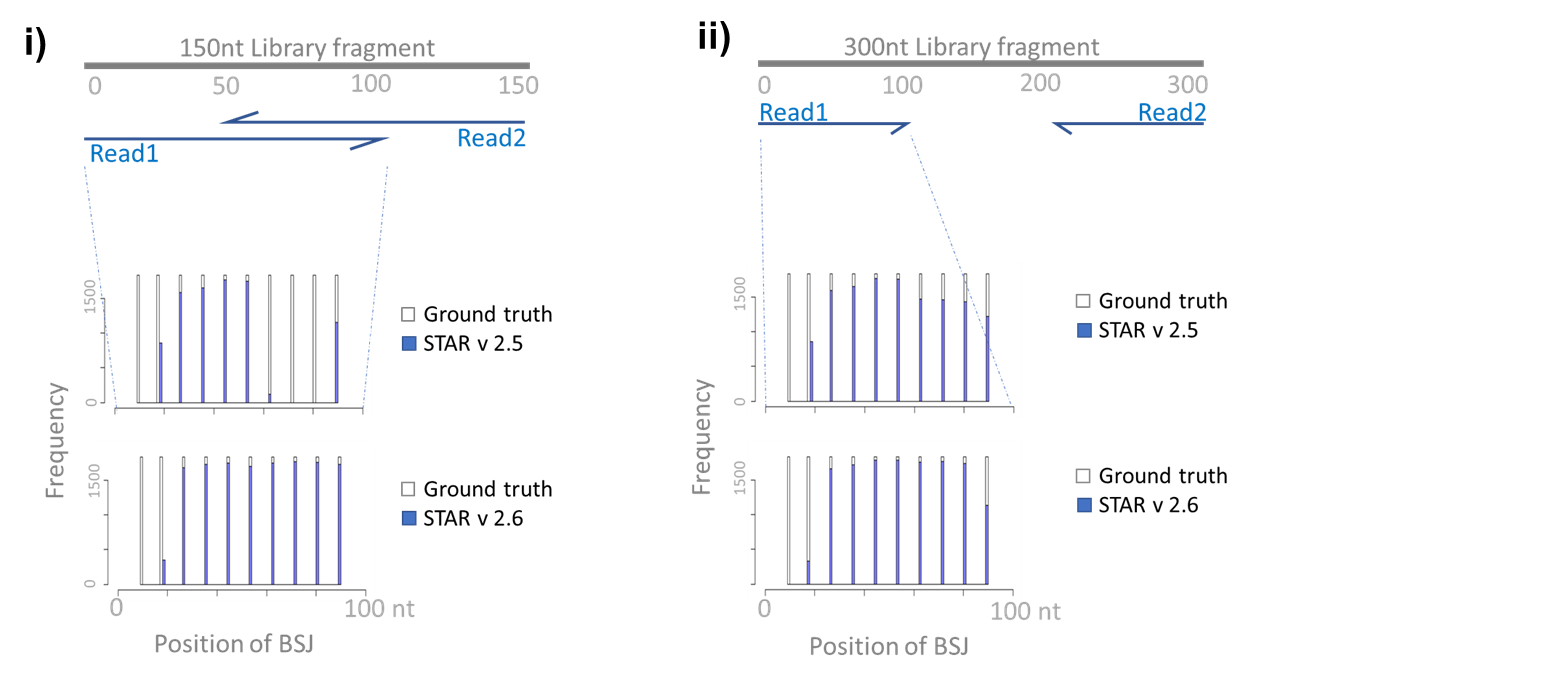


**Figure C:** Specificity of the STAR aligner in detecting BSJ in different library sized fragments. The ground truth was compared to different STAR aligner version in detecting BSJ. i) 150nt library fragments result in overlapping read pairs (assuming 100bp read length). STAR 2.5 fails to detect BSJ that are capture in both read pairs. ii) 300nt library fragments produce non-overlapping read pairs. Both versions of the STAR aligner detected the majority of BSJ, with version 2.6 performing slightly better.

**Sensitivity and specificity of the RAD and FSJ support score metrics**

The sensitivity and specificity of circRNA tools has been benchmarked in other studies on matched ribominus and RNaseR treated data sets [1, 2]. We used the same data sets to assess the performance of Ularcirc filter metrics (i.e. RAD score and FSJ support score) in discriminating true and false positives.

The RAD score was applied to BSJ having a read count greater than nine as significant skewing occurs at lower counts. Therefore, we also applied the same count threshold for circExplorer and CIRI outputs. Ularcirc BSJ data that had a RAD score between 0.1 and 0.9 identified 64% true positives which was one more than what was identified in circExplorer output (**Figure D i**). We noticed that a greater number of true positives were identified from BWA aligned data analysed with circExplorer and CIRI pipelines (**Figure D**), which has been previously described [3]. Ularcirc had a higher inclusion of false positives compared to CIRI and circExplorer pipelines (**Figure D**). Interestingly, in the list of false positives, we identified numerous lariat RNAs as well as BSJ that define circRNA validated in other studies – including the RNaseR sensitive circRNA CiRS-7. We suspect that these candidates are indeed true positives and are perhaps linearized versions of bona fide circRNA transcripts. It is therefore possible that other RNaseR sensitive candidates may also be derived from real circRNA.

We next considered using the FSJ support score (i.e. > 0) as a filter on BSJ having minimal counts as defined in previous analysis (i.e. 3 counts in at least one ribominus data set) [1, 2]. The FSJ support score filter retained 56% of true positives and included 28% false positives (**Figure D ii**). When applied to BSJ that have a count greater than 9, it had similar performance to the RAD score (**Figure D i**). More importantly, applying a filter that incorporates both the FSJ support score and the RAD score retained the majority of true positives and removed all but 15% of the false positives **(Figure D i)**.

Finally, we would like to emphasize that the Ularcirc workflow prioritises analysis on abundant BSJ candidates and is most time efficient when analysing subset of BSJ from either one or multiple data sets. When performing analysis, we recommend users to upload the gene counts, thereby enabling normalisation to total gene counts which is analogous to the “splice reads per billion mapping” (SRPBM) previously defined [4].


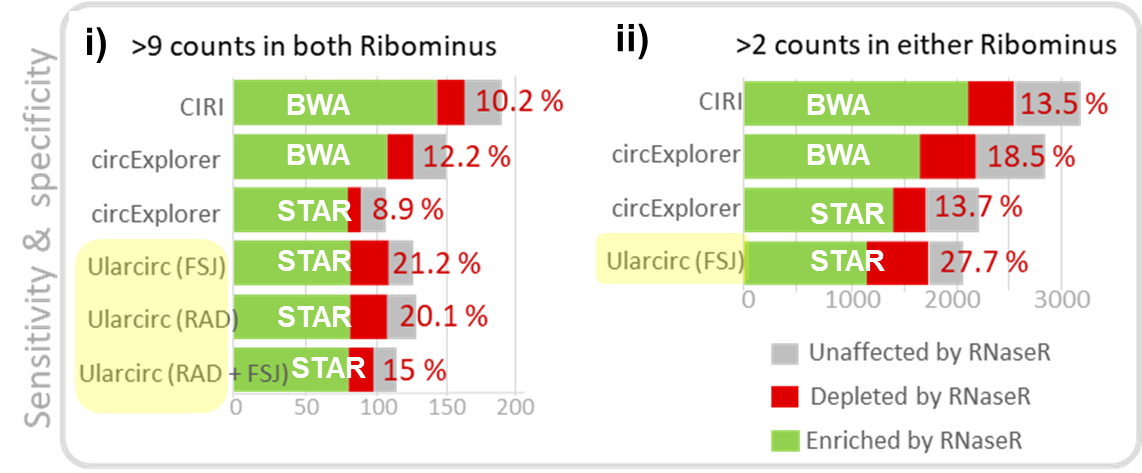


**Figure D** Sensitivity and specificity of Ularcirc, circExplorer and CIRI on two ribominus (SRR444655 and SRR444975) and two RNaseR (SRR444974, SRR445016) data sets, previously used to test circRNA software. Data sets were aligned with either BWA or STAR and then analysed with the listed circRNA software. Enriched BSJ are defined as those that have 5x more counts in RNaseR compared to ribominus datasets, while depleted BSJ are those that have no increase in counts. (i) Analysis of abundant BSJ having at least 9 counts in both ribominus data sets. (ii) Analysis of all BSJ that have at least 2 counts in either ribominus data set.

**Comparing Ularcirc to circExplorer2**

A review of five different software packages identified that each was capable of detecting a common pool of circRNA, but each software was also capable of detecting a unique set of true positive BSJ [1, 2]. The software package CircExplorer was considered as one of the best tools as it had high accuracy, good sensitivity and low memory consumption [2]. Furthermore, its successor, circExplorer2 can take inputs from a number of different aligners, including STAR, making it very versatile. We reanalysed data sets from Hs68 human fibroblasts [4] by aligning with the STAR aligner and compared the outputs of Ularcirc and circExplorer2 and found a high correlation in BSJ counts retrieved by both programs (**Figure E i**). All abundant BSJ identified by circExplorer were also identified by Ularcirc with the majority having identical counts (correlation = 0.916). This comparison also demonstrates that the default inbuilt RAD thesholds filter in Ularcirc does not filter out true circRNAs. Interestingly, a number of novel BSJ were identified specifically by Ularcirc. Closer examination revealed that circExplorer2 failed to identify some of these candidates because they did not overlap with known gene transcripts splice junctions, a known limitation of circExplorer. To demonstrate circExplorers dependency and limitations on using known gene models, we performed a theoretical exercise by re-analysing HEK293 RNase R treated RNA-Seq data sets [5]. We focused on ASAP1 gene which produces two alternative circRNA isoforms that use a common exon acceptor, as visualized with Ularcirc (**Figure E ii**). We modified the ASAP1 gene entry so that either the donor or acceptor exons that define a BSJ were shifted to either the 5’ or 3’ direction. Unsurprisingly, circRNA were not reported by circExplorer2 when either donor/acceptor exon was modified (**Figure E iii**). Ularcirc however was able to identify both circRNAs without a gene reference.


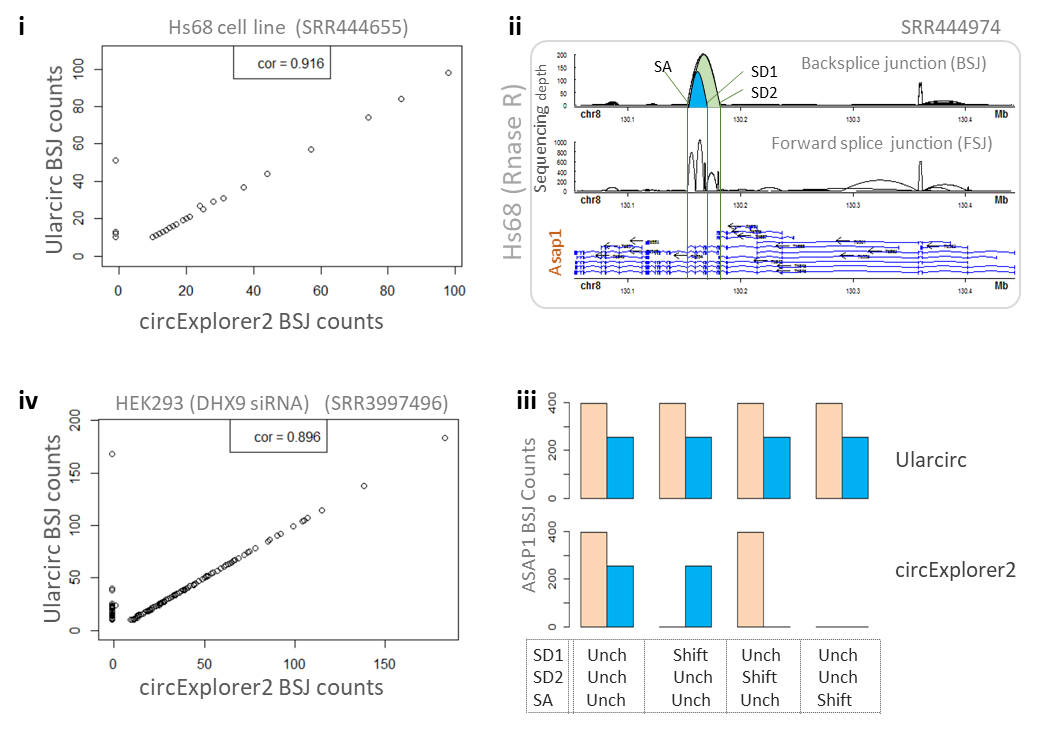


**Figure E** Ularcirc identification of BSJ is superior to circExplorer2. i) Comparison of BSJ raw counts recovered from Ularcirc and circExplorer2. ii) Two Asap1 circRNA share a common splice acceptor (SA) but originate from two different splice donors (SD1, SD2). iii) Demonstrating the limitation of using gene model-dependent circRNA discovery. Gene entries for *ASAP1* were either left unchanges (Unch) or the splice sites listed in (ii) were shifted 5’ and 3’ (shift) by 10nt before running the various software. CircExplorer2 fails to detect the circRNAs when corresponding *ASAP1* gene exons boundaries are shifted. Ularcirc identify both circRNAs even if the gene models are altered. iv) Ularcirc identifies abundant circRNA from *DHX9* knockdown cells which were previously missed due to the use of circExplorer2.

**Comparing internal splicing of circRNAs with Ularcirc and CIRI-AS**

The circRNA analysis tool CIRI-AS is capable of identifying internal splicing patterns within circRNA. CIRI-AS implements this by quantifying FSJs that are captured on the same library fragment as a corresponding BSJ. FSJ not associated with BSJ are not quantified and therefore this analysis limits the detection of internalised splicing to the fragment length of the sequencing platform.

Ularcirc quantifies FSJ that we classify as internal, external or spaning a BSJ (**Figure F i and Supplementary Figure 6**). At this point in time, Ularcirc does not quantify FSJ that are captured on BSJ library fragments, and therefore a global comparison to CIRI-AS is not possible. We did identify some data sets that revealed the internal splicing patterns of circRNA (refer **Figure 5**). The most striking are RNAse R treated data sets where linear isoforms are degraded and the remaining FSJ are internal to the detected BSJ. For these data sets users can download/view FSJ coordinate tables on the gene view tab which would represent the internal structure of the selected circRNA.

We also demonstrated that analysis of time course data sets can reveal internal splicing of upregulated circRNA (**Figure 5A**). Because this observation is not conclusive, we decided to cross examine data sets with CIRI-AS. CIRI-AS confirmed the boundaries of two exons which matched perfectly with the coordinates of the upregulated FSJ as seen using Ularcirc (**Figure 6** inset). The remaining internal exons junctions were not reported by CIRI-AS as they extended outside the length of standard library preps.


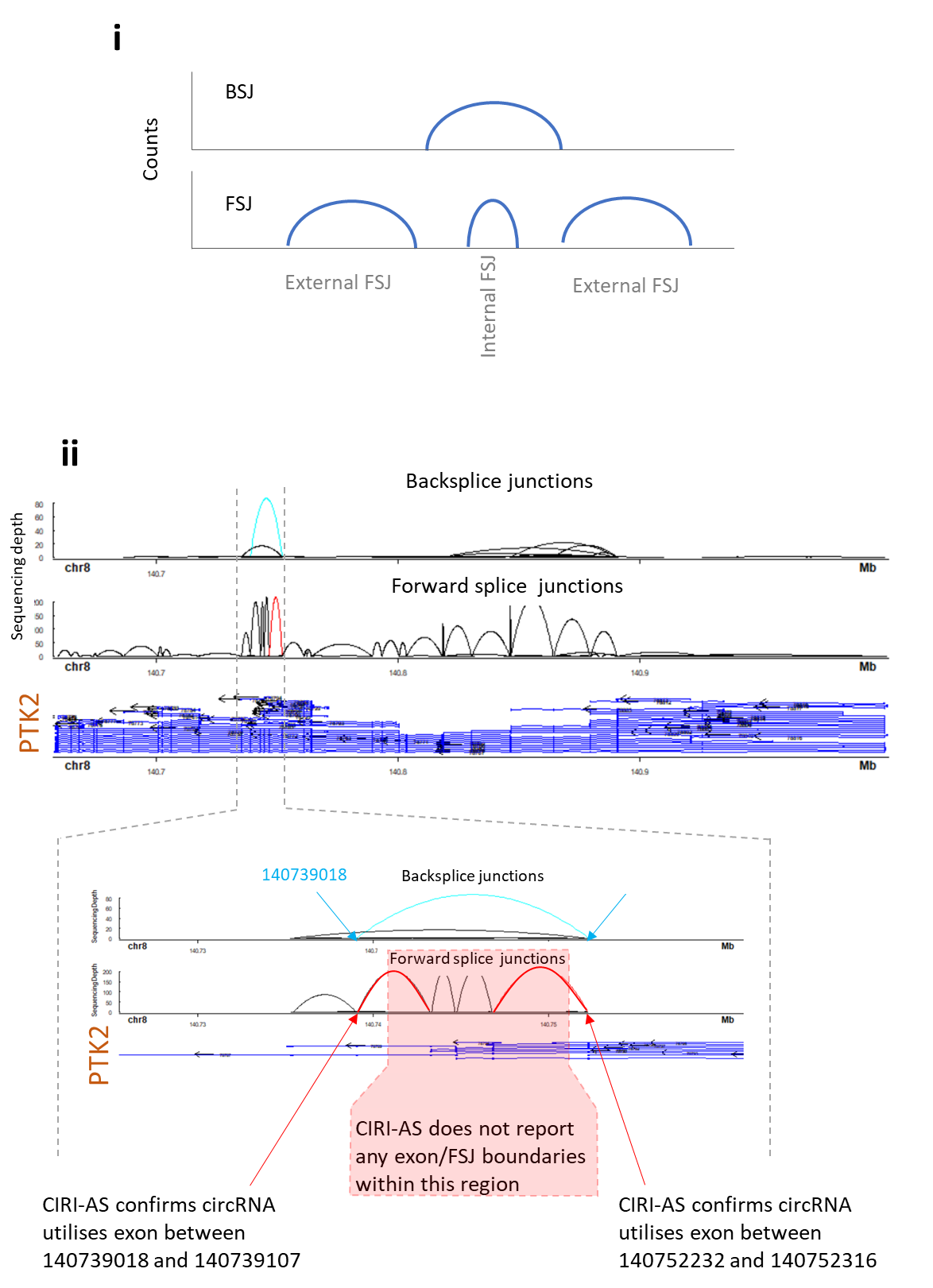


**FIGURE F**: i) Breakdown of FSJ classification, external and internal FSJ are named relative to their position of the corresponding BSJ. ii) Examination of internal FSJ of circRNA from PTK2. Ularcirc identified four internal FSJ of circRNA PTK2. The same data set was analysed by CIRI-AS and reported the outer most exons (between BSJ coordinates and the red FSJ).

**Ularcirc running metrics**

Ularcirc is a versatile software package that can assemble junction counts from a variety of sources. For pre-assembled count tables Ularcirc is very efficient in collating complete data sets, generating count tables, preparing visualisations. However utilising Ularcircs inbuilt circRNA discovery pipeline on chimeric junction tables from the STAR aligner is computationally expensive and requires some time to complete. Currently Ularcirc is implemented to use one thread and we envision that users will prioritise analysis on 10 – 100 BSJ. Table 1 below highlights the time taken to build a grouped BSJ table from the “TwoSzabo” dataset as provide with the software with different annotation options.

**Table A**: Running time for Ularcirc to assemble tables of BSJ counts and annotations from STAR output.

| TwoSzabo data sets | No annotation | Parental gene annotation | Internal/external/spaning FSJ annotation |
| --- | --- | --- | --- |
| Requested top 10 BSJ/sample  (total 19 BSJ entries returned) | <2s | < 5s | <35s |
| Requested top 20 BSJ/sample  (total 37 BSJ entries returned) | < 3s | < 8s | < 60s |
| Requested top 100 BSJ/sample  (176 BSJ entries) | <5s | < 30s | <250s |

**Bibliography**

1. Hansen TB: **Improved circRNA Identification by Combining Prediction Algorithms.** *Front Cell Dev Biol* 2018, **6:**20.

2. Hansen TB, Veno MT, Damgaard CK, Kjems J: **Comparison of circular RNA prediction tools.** *Nucleic Acids Res* 2016, **44:**e58.

3. Gao Y, Zhao F: **Computational Strategies for Exploring Circular RNAs.** *Trends Genet* 2018, **34:**389-400.

4. Jeck WR, Sorrentino JA, Wang K, Slevin MK, Burd CE, Liu J, Marzluff WF, Sharpless NE: **Circular RNAs are abundant, conserved, and associated with ALU repeats.** *RNA* 2013, **19:**141-157.

5. Aktas T, Avsar Ilik I, Maticzka D, Bhardwaj V, Pessoa Rodrigues C, Mittler G, Manke T, Backofen R, Akhtar A: **DHX9 suppresses RNA processing defects originating from the Alu invasion of the human genome.** *Nature* 2017, **544:**115-119.
